# The costs of delivering vaccines in low- and middle-income countries: Findings from a systematic review

**DOI:** 10.1016/j.jvacx.2019.100034

**Published:** 2019-07-15

**Authors:** Kelsey Vaughan, Annette Ozaltin, Michaela Mallow, Flavia Moi, Colby Wilkason, Juliana Stone, Logan Brenzel

**Affiliations:** a ThinkWell, 1875 Connecticut Avenue, 10th Floor, Washington, DC 20009, USA; b Bill & Melinda Gates Foundation, 440 5th Ave N., Seattle, WA 98109, USA

**Keywords:** Costs and cost analysis, Systematic review, Vaccines, Immunization delivery, Immunization financing, Low- and middle-income countries

## Abstract

**Introduction:**

Information on immunization delivery costs (IDCs) is essential for better planning and budgeting for the sustainability and performance of national programs. However, delivery cost evidence is fragmented and of variable quality, making it difficult for policymakers, planners, and other stakeholders to understand and use. This study aimed to consolidate and summarize the evidence on delivery costs, answering the question: What are the unit costs of vaccine delivery across low- and middle-income countries (LMICs) and through a variety of delivery strategies?

**Methods:**

We conducted a systematic review of over 15,000 published and unpublished resources from 2005 to 2018 that included IDCs in LMICs. We quality-rated and extracted data from 61 resources that contained 410 immunization delivery unit costs (e.g., cost per dose, cost per fully immunized child). We converted cost findings to a common year (2016) and currency (U.S. dollars) to ensure comparability across studies and settings. We performed a descriptive and gap analysis and developed immunization delivery cost ranges using comparable unit costs for single vaccines and schedules of vaccines.

**Results:**

The majority of IDC evidence comes from low-income countries and Sub-Saharan Africa. Most unit costs are presented as cost per dose and represent health facility-based delivery.

**Discussion:**

The cost ranges may be higher than current estimates used in many LMICs for budgeting: $0.16–$2.54 incremental cost per dose (including economic, financial, and fiscal costs) for single, newly introduced vaccines, and $0.75–$9.45 full cost per dose (economic costs) for schedules of four to eight vaccines delivered to children under one.

**Conclusions:**

Despite increased attention on improving coverage and strengthening immunization delivery, evidence on the cost of delivery is nascent but growing. The cost ranges can inform planning and policymaking, but should be used with caution given their width and the few unit costs used in their development.

## 1 Introduction

As low-and middle-income countries (LMICs) drive toward achieving high and equitable coverage of life-saving vaccines and largely transition from donor- to self-funded immunization programs, the availability of sufficient, sustainable, equitable, and predictable financing for vaccine delivery is essential. Such financing is built on solid evidence about the costs of delivering immunization services (sometimes called delivery or operational costs), but cost data are often fragmented, of variable quality, and are difficult for policymakers, program planners, and other global and country-level stakeholders to understand and use in their own settings [1]. Further, conducting country-specific costing studies can be time consuming and expensive.

Immunization delivery cost (Box 1) evidence that is reliable and easily accessible can help countries better advocate for additional resources, plan and budget, and make programmatic decisions. Past systematic reviews on immunization delivery costs (IDCs) have consolidated only a portion of the costing evidence. They focused on either (1) a subset of vaccines, (2) a subset of economic evaluations (e.g., only cost-effectiveness or cost-benefit studies), or (3) only the incremental costs of new vaccine introduction (NUVI) [2], [1], [3], [4].

Box 1 **Definition of Immunization Delivery Costs (IDC).**

We define immunization delivery costs (IDCs) (also referred to as operational costs) as the costs associated with delivering immunizations to target populations, exclusive of vaccine costs. Delivery costs may include any or all of the following recurrent and capital cost items: (1) paid human resources, (2) volunteer human resources, (3) per diem and travel allowances, (4) cold chain equipment and their overheads (e.g. energy, maintenance, repairs), (5) vehicles, transport and fuel, (6) program management, (7) training and capacity building, (8) social mobilization and advocacy, (9) disease surveillance and activities related to adverse events following immunization (AEFI), (10) buildings, utilities, other overheads and shared costs, (11) vaccine supplies (e.g. safety boxes, diluents, reconstitution syringes), (12) waste management, (13) other supplies and recurrent costs, and (14) other non-vaccine costs.

Definition is based on [5], [21], and [6].

Additionally, the large number of recently published articles on IDCs reflects a need to bring the evidence base up to date, standardize it to the extent possible, and make it accessible and easy to use by national and sub-national planners, policymakers, researchers and international partners for policy advocacy, planning, research and other related efforts. We embarked upon this systematic review as part of the Immunization Costing Action Network (ICAN) – a research and learning community that aims to increase the visibility, availability, understanding, and use of evidence on the cost of delivering vaccines [7].

## 2 Methods

We conducted a systematic review of published articles and grey literature (resources) spanning from 2005 to 2018 on IDCs in LMICs, and developed a repository where cost and other information reported in the resources can be accessed. We also assessed the data for spread/scope of the evidence, methods/reporting of the costing studies, and quality of the resources. We developed immunization delivery cost ranges (cost ranges) for delivery of specific vaccines or types of vaccines, by different delivery strategies, and for different country income levels and regions. Methods and results were subject to external review and revision by an international advisory group of immunization costing experts.

### 2.1 Search strategy and screening

In January 2017 and again in April 2018, we searched six electronic databases – EconLit, Embase, Medline (via PubMed), NHS-EED, Web of Science, and WHO Global Index Medicus – for peer-reviewed articles published between January 2005 and January 2017, and later January 2017 and April 2018, that included IDCs for all countries of any income level. We did not go further back than 2005 in order to reflect current vaccine delivery technologies and established costing methods for the sake of greater comparability, and to limit the size of the search. Search terms included three categories of keywords – “immunization” AND “cost” AND “delivery” – and were translated into the query language of each database. Supplementary Appendix 1 presents the database queries and resulting yields. In addition, we reviewed reference lists of all articles, plus references used in systematic reviews.

To capture unpublished reports, we sent direct requests to 64 key contacts at organizations involved in global and national immunization-related work. In addition, we posted a call for grey literature in eight immunization newsletters, communities of practice and web discussion forums. We applied advanced search syntax in Google to search for resources on the webpages of key organizations and relevant databases housed within these organizations. We also searched conference proceedings and the ProQuest dissertation database. These searches used terms to capture resources relevant to immunization delivery and costs. Actual strategies used in searches varied by the organizations and forums targeted, and were refined iteratively.

Following the search, we removed duplicate resources, clearly irrelevant resources (i.e., veterinary, in vitro, high-income country, qualitative and therapeutic studies), and resources published before 2005. We removed systematic reviews after examining reference lists to ensure we had captured relevant secondary resources to screen for our review.

We included resources with full text availability in English, French, or Spanish that reported immunization delivery unit costs (i.e., cost per dose, per capita, per full immunization of a vaccine, per fully immunized child, and per person in the target population). Full immunization of a vaccine refers to all required doses of a specific vaccine (e.g., two doses of oral cholera vaccine (OCV)). Fully immunized child refers to the provision of required vaccines to a specific group by a clear point in time (e.g., infants who received all vaccines in the schedule before reaching one year of age). We used the resource authors’ definition of fully immunized child that was relevant for their studies, as opposed to a standard global definition, for example, of DTP3.

We included resources reporting costing, cost-effectiveness, cost-benefit, return on investment, cost-utility, and other analyses that included unit cost data. We included only those resources reporting IDCs in LMICs, with country income levels determined using the World Bank classification [8].

We excluded resources if they used secondary or modeled unit cost estimates alone or if the costing methodology was unclear or insufficient to allow for extraction and analysis. In the case of unclear/insufficient methods but where all other inclusion criteria were met, we contacted authors of those resources and the resource was included if the necessary information was obtained.

Four investigators completed title, abstract, and full text review according to standard methods [9]. Two investigators reviewed title and abstract exclusions, with disagreements resolved by consensus.

### 2.2 Data extraction and cleaning

Resources meeting all inclusion criteria after title, abstract and full text review underwent data extraction. We extracted information on the context of country and study, details of the study design and costing methodology, the vaccines costed and their delivery strategies, the cost categories included, and the reported results. We extracted data as reported by the authors without any recoding or analysis, but we noted where the reported methods (e.g., study perspective) appeared to deviate from commonly accepted definitions [10].

We entered extracted data into a Microsoft Excel data extraction tool. We designed the data extraction tool using an iterative approach, piloting it on three resources, and thereafter revising it. We conducted preliminary data analyses over a two-week period to evaluate the tool’s design and ensure inter-extractor reliability, with subsequent further revisions. Two investigators reviewed full text extractions, returning incomplete extractions to the original investigator extracting the data for any necessary revisions.

We cleaned the data to ensure consistency across extractions and to allow for further analysis. We also added contextual information, such as region and country income level, using World Bank country classifications [8].

### 2.3 Quality assessment

We assessed the quality of each resource using a set of quality criteria designed for this review, building on other quality assessment systems and checklists [11], [12], [13], [14], [15], [16], [10]. We grouped the quality assessment criteria into three categories: methodological rigor and reporting standards (8 items), uncertainty of results (3 items), and risk of bias and limitations (3 items). For each resource, each item was given an individual score of 1 (lowest), 2, or 3 (highest); for some items there was also a “not applicable” option. Scores for all items were summed and averaged, excluding any “not applicable” answers, to produce a final score for each resource on the 1–3 scale. Supplementary Appendix 2 provides a full description of the quality assessment methodology.

### 2.4 Conversion of cost findings

We converted all cost findings to 2016 US dollars (US$) to ensure comparability across currencies and time. The chosen methodology was decided in consultation with five immunization costing experts as the best way to account for local currency unit (LCU) inflation and LCU-US$ currency exchange fluctuations. For costs reported in US$, we first converted costs to LCUs of the same year using the exchange rate at the year of costing, or the year of data gathering if the year of costing was not reported. If neither of these two were available, we used the publication year of the resource. Exchange rates were taken from the World Bank (‘US$ per LCU, period average’) [17]. With the costs reported in US$ now in LCUs, and for costs originally reported in LCUs by the resource, we inflated costs to 2016 LCUs using LCU inflation rates reported by the International Monetary Fund (‘inflation, average consumer prices’) [18]. With all costs in 2016 LCUs, we converted costs to 2016 US$ using the ‘LCU per US$, period average’ official exchange rate for 2016 [17].

### 2.5 Building a cost repository

To house both the extracted data and standardized data in 2016 US$, we developed the Immunization Delivery Cost Catalogue (IDCC) – a web- and Excel-based cost repository. Both versions include filtering and data search functionality, enabling the user to select specific data criteria to view and analyze. After several rounds of user testing, we made the IDCC publicly available at http://immunizationeconomics.org/ican-idcc.

## 3 Data analysis

### 3.1 Descriptive and gap analysis

We analyzed the spread/scope of the evidence, the methods/reporting of the included studies, and the quality of the extracted resources. We used Excel to run basic counts of the unit cost dataset for different criteria (e.g., unit costs reporting economic costs, unit costs including paid human resources as a cost category, etc.). Based on these counts, we described the evidence and identified gaps.

### 3.2 Cost ranges

From the cost catalogue data, we created immunization delivery cost ranges (cost ranges) for delivery of specific vaccines or types of vaccines, by different delivery strategies, and for different country income levels and regions. We applied seven mandatory comparability criteria to first identify unit costs from different resources that are methodologically and contextually similar, considering the type of cost (e.g., economic, financial, or fiscal; incremental or full), delivery platform and scale (routine vs. supplementary immunization activity [SIA]; pilot/project or full) and other factors (Box 2). We then checked for comparability of these unit costs against a set of additional methods criteria as well as vaccine delivery and country contextual information. These criteria were not used to develop the cost ranges, but were considered in the judgment of the validity of the cost range, which may have resulted in the removal of a unit cost from the range. Common reasons for the lack of comparability of unit costs were their different delivery platforms (i.e., routine vs. SIA), or different level of costs included (i.e., national vs. pilot/project). For example, we did not use unit costs of delivering OCV through a campaign in a cost range with those of introducing HPV in schools as part of a demonstration project.

Box 2 **Unit cost comparability criteria for cost ranges.**

Mandatory comparability criteria:

• Economic, financial, or fiscal costs (see Box 3)
• Full or incremental costing.
• Introduction/startup and/or recurrent/ongoing costs.
• Highest level of costs included.
• Supply chain only costs.
• Delivery platform (routine vs. SIA)
• Delivery scale (pilot/project or full)

Additional comparability criteria – methods:

• Number of sampled facilities.
• Perspective.
• Number of included cost categories (of 14 total)
• Important cost categories included (paid human resources; cold chain equipment and their overheads; vehicles, transport and fuel; training and capacity building)

Additional comparability criteria – vaccine delivery:

• Vaccines costed
• Number of antigens costed.
• For single vaccines: mode of administration (oral, injectable)
• For multiple vaccines: number of contacts required with the health system.
• Target delivery population.
• New vaccine introduction status.
• Vaccine delivery strategy (e.g., health facility, school, outreach, mobile, campaign)
• Delivery sector (e.g. public, NGO, etc.)

Additional comparability criteria – context:

• Country and number of countries used.
• Region.
• Country income level.
• Population size.
• Population density.
• Geographic setting.

For combinations of four or more comparable unit costs, we developed cost ranges and associated descriptive statistics (i.e., minimum, maximum, median, mean, and 25th and 75th percentile values), with accompanying visuals and methodological notes to facilitate interpretation.

## 4 Results

### 4.1 Search and review results

**Fig. 1:**
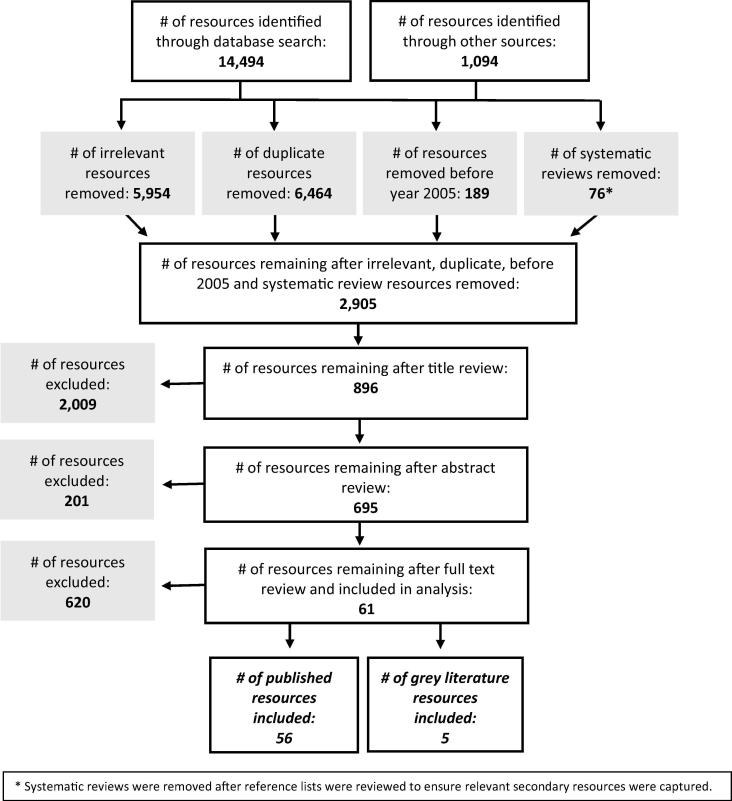
Review process of resources on immunization delivery costs.

From 15,588 initial resources, a total of 2,905 resources underwent title, abstract, and/or full text review (Fig. 1). Ultimately, we extracted data from 61 resources: 56 from peer-reviewed publications and five from grey literature. Supplementary Appendix 3 lists the resources included.

From the 61 resources included in the systematic review, we extracted 410 immunization delivery unit costs, which are included in the web and Excel IDCC, presented in 2016 US$, along with relevant explanatory information on the methodology and costing study context.

### 4.2 Descriptive analysis results

**Fig. 2:**
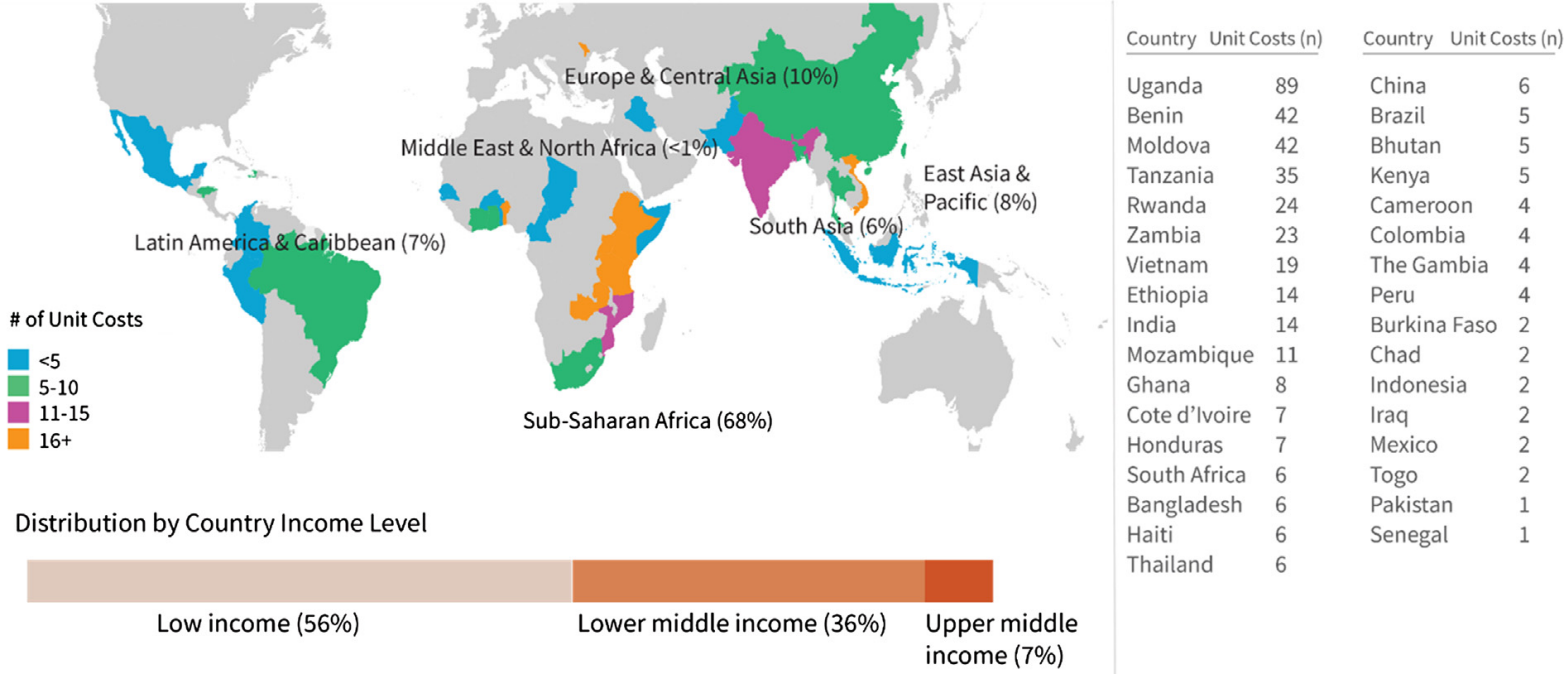
Geographic spread of immunization delivery unit costs.

The majority (70%) of immunization delivery unit costs come from resources published from 2014 through 2016. Only 6% of unit costs come from resources published from 2005 through 2009. Nearly half (45%) of the unit costs come from the EPI Costing and Financing Project (EPIC), a multi-country immunization costing and financing project supported by the Bill & Melinda Gates Foundation [19]. Immunization delivery unit cost data is available from a total of 33 LMICs (Fig. 2). The largest number of unit costs (22%) come from Uganda. Six countries (Benin, Moldova, Rwanda, Tanzania, Uganda and Zambia) contributed 62% of the unit costs in the IDCC. For eight countries (Burkina Faso, Chad, Indonesia, Iraq, Mexico, Pakistan, Senegal and Togo), only one or two immunization delivery unit costs are available.

The Sub-Saharan Africa region accounts for the majority (68%) of immunization delivery unit costs, followed by the Europe and Central Asia region (10%). The remaining 22% of immunization delivery unit costs come from East Asia and Pacific (8%), Latin America and the Caribbean (7%), South Asia (6%) and the Middle East and North Africa (<1%) (Fig. 2).

More than half of the immunization delivery unit costs come from low-income countries, and more than one-third come from lower-middle income countries. The remaining 7% of unit costs come from upper-middle-income countries (Fig. 2).

**Fig. 3:**
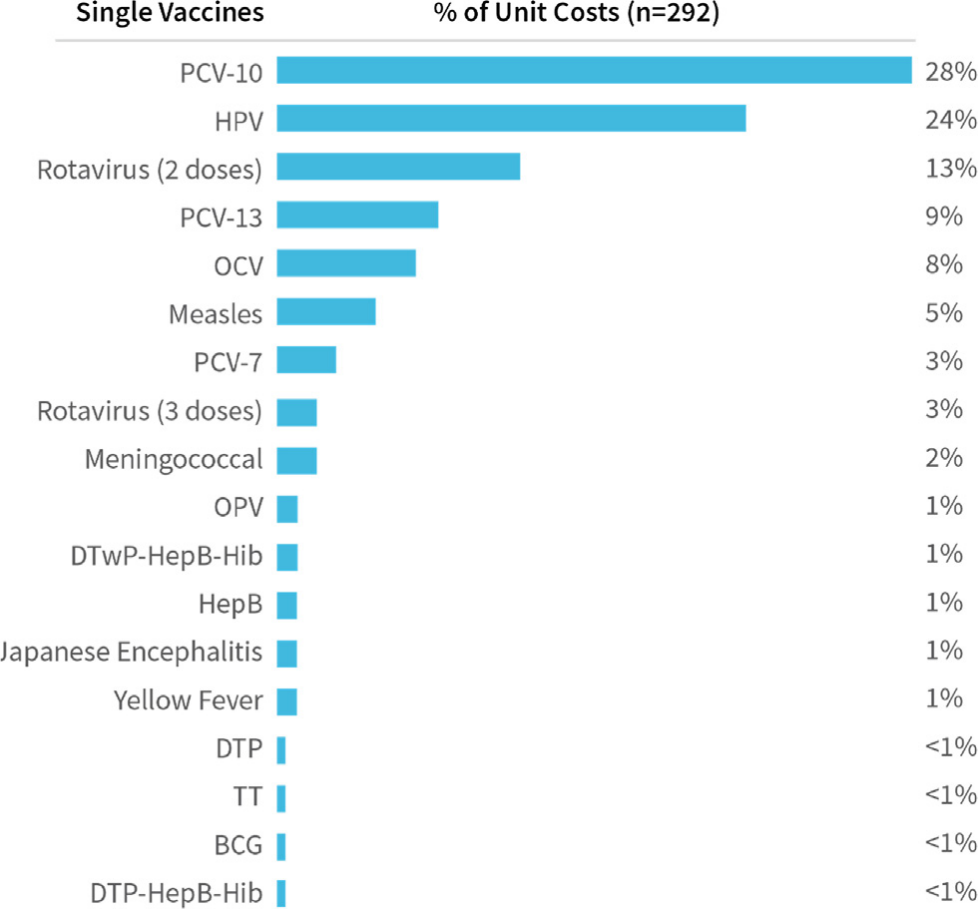
*Distribution of immunization delivery unit costs for single vaccines.* *Notes:* Percentages do not sum to 100% due to rounding. There were no articles with immunization delivery unit cost estimates for Inactivated Poliovirus Vaccine (IPV). *Codes:* BCG = Bacillus Calmette-Guérin; DTP = Diphtheria and tetanus toxoids and whole-cell pertussis vaccine, pediatric formulation; HepB = Hepatitis B; Hib = Haemophilus influenzae type b; HPV = Human Papillomavirus; JE = Japanese Encephalitis; OCV = Oral Cholera Vaccine; OPV = Oral Polio Vaccine; PCV = Pneumococcal Conjugate Vaccine (7-, 10-, or 13-valent); TT = Tetanus Toxoid.

More than two-thirds (71%) of unit costs in the dataset are for single vaccines only, with the remainder associated with delivery of more than one vaccine or a schedule of vaccines. Of the single vaccine unit costs, 84% are in the context of new vaccine introduction. Most single vaccine unit costs are for PCV7/10/13 (40%), HPV (24%) and Rotavirus (2- and 3-doses) (16%) (Fig. 3).

**Fig. 4:**
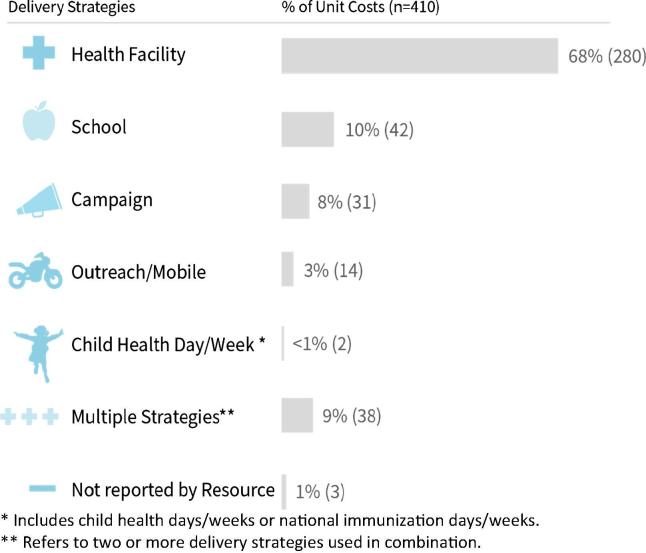
Immunization delivery unit cost data availability by delivery strategy.

Most of the immunization delivery unit costs (68%) pertain to health facility delivery, reflecting the predominant delivery of vaccines at fixed sites. There are also a substantial number (10%) of unit costs for school-based delivery, all published since 2010 and focused on HPV introduction (Fig. 4).

Over half (63%) of the immunization delivery unit costs relates to new vaccine introduction, of which 83% are costed incrementally. Of the unit costs for new vaccine introduction, 45% report the costs of introducing PCV (7/10/13), and 27% report the costs of introducing HPV.

Most immunization delivery unit costs (80%) note the perspective taken; the largest share of unit costs is from costing studies that took a government (44%) or provider (30%) perspective.

Comparing incremental and full costing, 60% of the unit costs are incremental, 34% are full costs for routine immunization delivery, and 6% are either unclear or not reported in the resource. Almost half (49%) of the immunization delivery unit costs represent economic costs, 32% represent financial costs, 11% represent fiscal costs, and the type of costs for the remaining 7% is either unclear or not reported in the resource (Box 3 and Table 1).

Box 3 **Definitions of economic, financial, and fiscal costs.**

• Economic costs: Financial outlays plus opportunity costs of health worker time and any donated items such as vaccines.
• Financial costs: Financial outlays, usually with straight-line depreciation of capital items.
• Fiscal costs: financial outlays, usually without depreciation of capital items.

Definitions are based on [5].

**Table 1:** Immunization delivery unit cost data availability by costing type.

Type of Costing	Number of Unit Costs by Type (% of Total)				Total Unit Costs
	Economic	Financial	Fiscal	Not reported/unclear	
Full	87 (21%)	29 (7%)	0 (0%)	18 (4%)	140 (34%)
Incremental	108 (26%)	84 (20%)	46 (11%)	8 (2%)	246 (60%)
Not reported	4 (1%)	20 (5%)	0 (0%)	0 (0%)	24 (6%)
Total Unit Costs	199 (49%)	133 (32%)	46 (11%)	26 (6%)	410 (100%)

*Note:* Percentages do not sum to 100% due to rounding.

Across all types of unit costs (i.e., economic, financial, and fiscal), the majority include the key immunization delivery cost drivers, namely: vehicles, transport, and fuel (included in 98% of the unit costs); cold chain equipment and overheads (88%); paid human resources (80%); and training and capacity building (77%) (Supplementary Appendix 4). However, there are differences by unit cost type. For example, financial costing included the following more frequently than economic costing studies: adverse event monitoring; program management; social mobilization and advocacy; training and capacity building; vaccine supplies; and waste management. Overall, volunteer human resources was the least reported cost category, though as expected, economic costing studies included volunteer human resources more frequently than financial or fiscal costing studies.

More than half of the cost categories (8 of 14 cost categories) are included in 82% of the immunization delivery unit costs, with 10 or more cost categories included in 51% of the unit costs. Fourteen percent of the unit costs include supply chain-only related costs (3–6 cost categories of 14).

### 4.3 Quality assessment

The overall mean quality score across the resources was 2.2 out of 3.0. The two assessment categories with the highest mean score were “Contextual factors: are there any contextual factors related to the study setting that have not been accounted for in the results?” (2.9/3.0), meaning that all resources that reported contextual factors took them into account in the results, and “Replicability: was the purpose of the study clearly defined?” (2.9/3.0). The category with the lowest score was “Data analysis strategy: were statistical tests used and confidence intervals reported?” (1.1/3.0), indicating that the overwhelming majority of resources did not report sufficient methodological detail in this area. A category that also scored low (1.3/3.0) was “Sensitivity analysis: if done, did the sensitivity analysis include all reasonable scenarios affecting costing results?” Quality assessment scores for all resources are accessible in the web- and Excel-based cost repositories, at http://immunizationeconomics.org/ican-idcc.

### 4.4 Cost ranges

We explored over 14,000 combinations of unit costs to identify those that are comparable. This exploration resulted in over 300 unique combinations of unit costs that we checked for comparability using specific criteria (Box 2). From these attempts, we identified eight sets of comparable unit costs, presented here as cost ranges. For each range we identify whether the unit costs are economic, financial and/or fiscal, whether full or incremental, as well as the relevant delivery strategy.

**Fig. 5:**
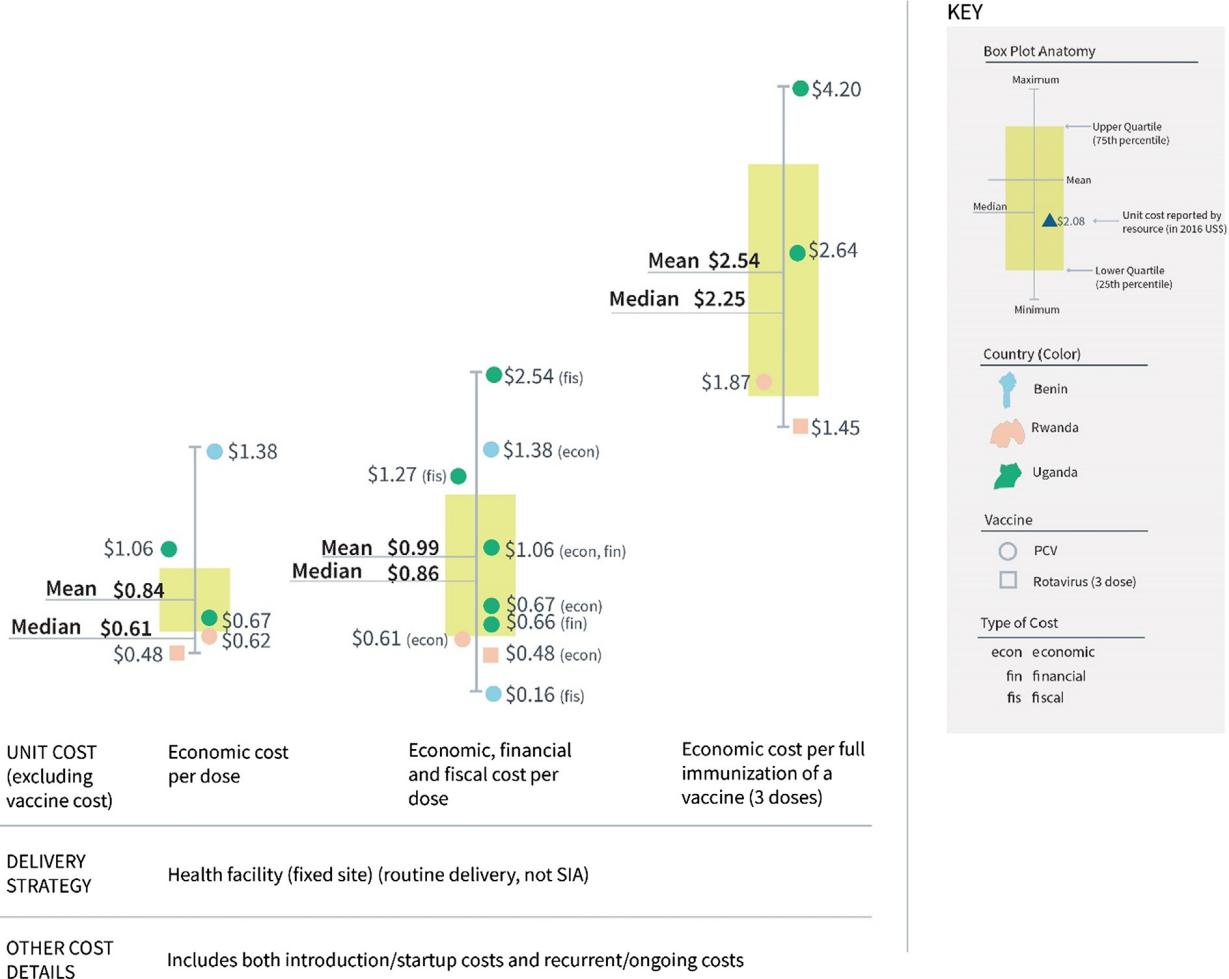
Incremental cost range for single, newly introduced vaccines, excluding vaccine cost (2016 US$).

In low-income countries, the incremental cost per dose (excluding vaccine cost) to deliver single, newly introduced vaccines at health facilities ranged from $0.48 to $1.38 considering only economic costs, with a mean of $0.84 and a median of $0.61 (Fig. 5, left boxplot). This contrasts with a range from $0.16 to $2.54 considering economic, financial and fiscal costs, with a mean of $0.99 and a median of $0.86 (Fig. 5, middle boxplot). The incremental cost for full immunization (i.e., 3 doses) of a vaccine ranged from $1.45 to $4.20 considering only economic costs, with a mean of $2.54 and a median of $2.25 (Fig. 5, right boxplot). (See also Supplementary Appendix 5).

**Fig. 6:**
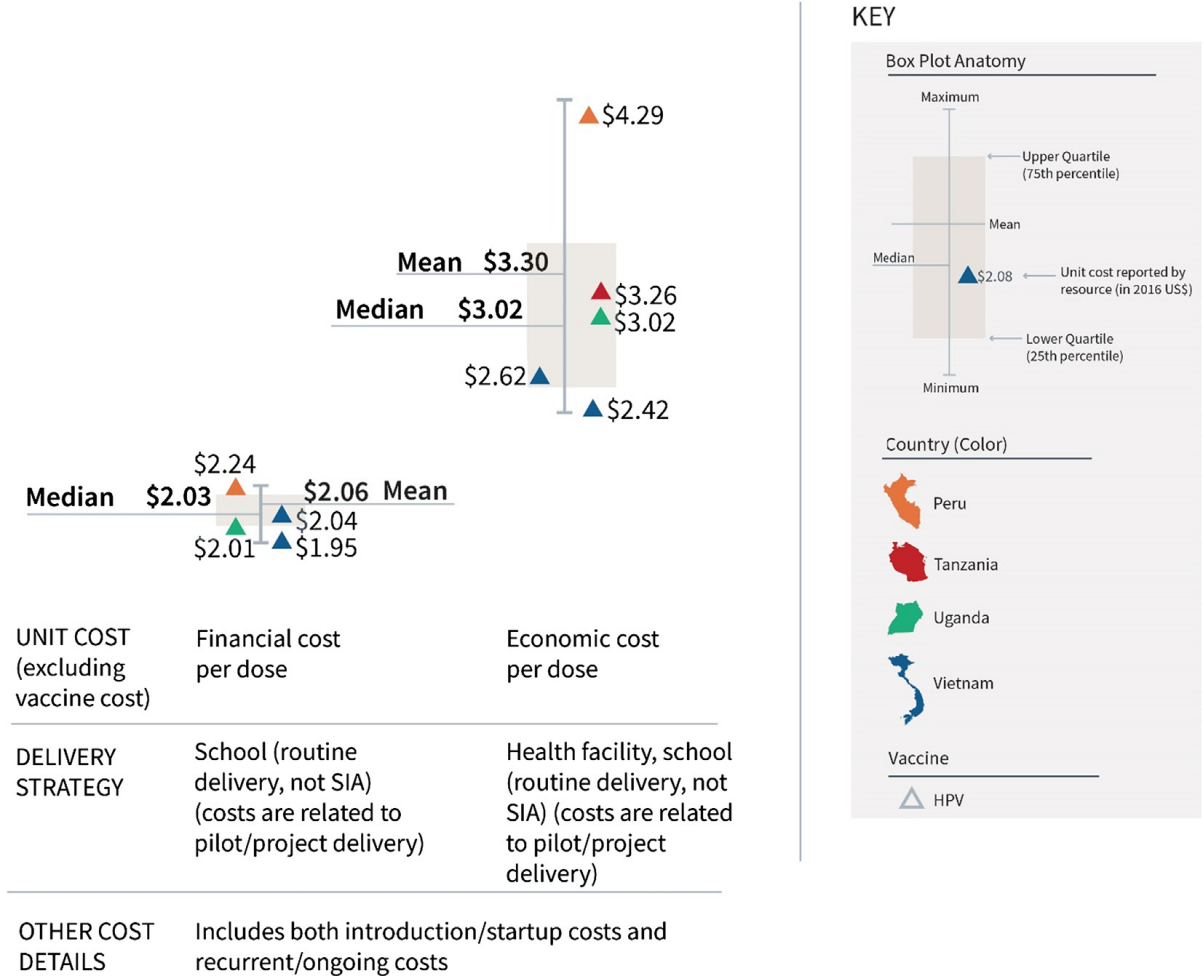
Incremental cost range for introducing HPV vaccine to an existing schedule, excluding vaccine cost (2016 US$).

The incremental costs of introducing HPV vaccine via school- and health facility-based delivery on a pilot/project basis (excluding vaccine cost) ranged from $1.95 to $4.29 per dose (Fig. 6). The lower end of the range represents financial costs for school-based delivery (left boxplot; mean $2.06 and median $2.03), whereas the higher end of the range corresponds with economic costs for health facility and school-based delivery (right boxplot; mean $3.30 and median $3.02). (See also Supplementary Appendix 6.)

**Fig. 7:**
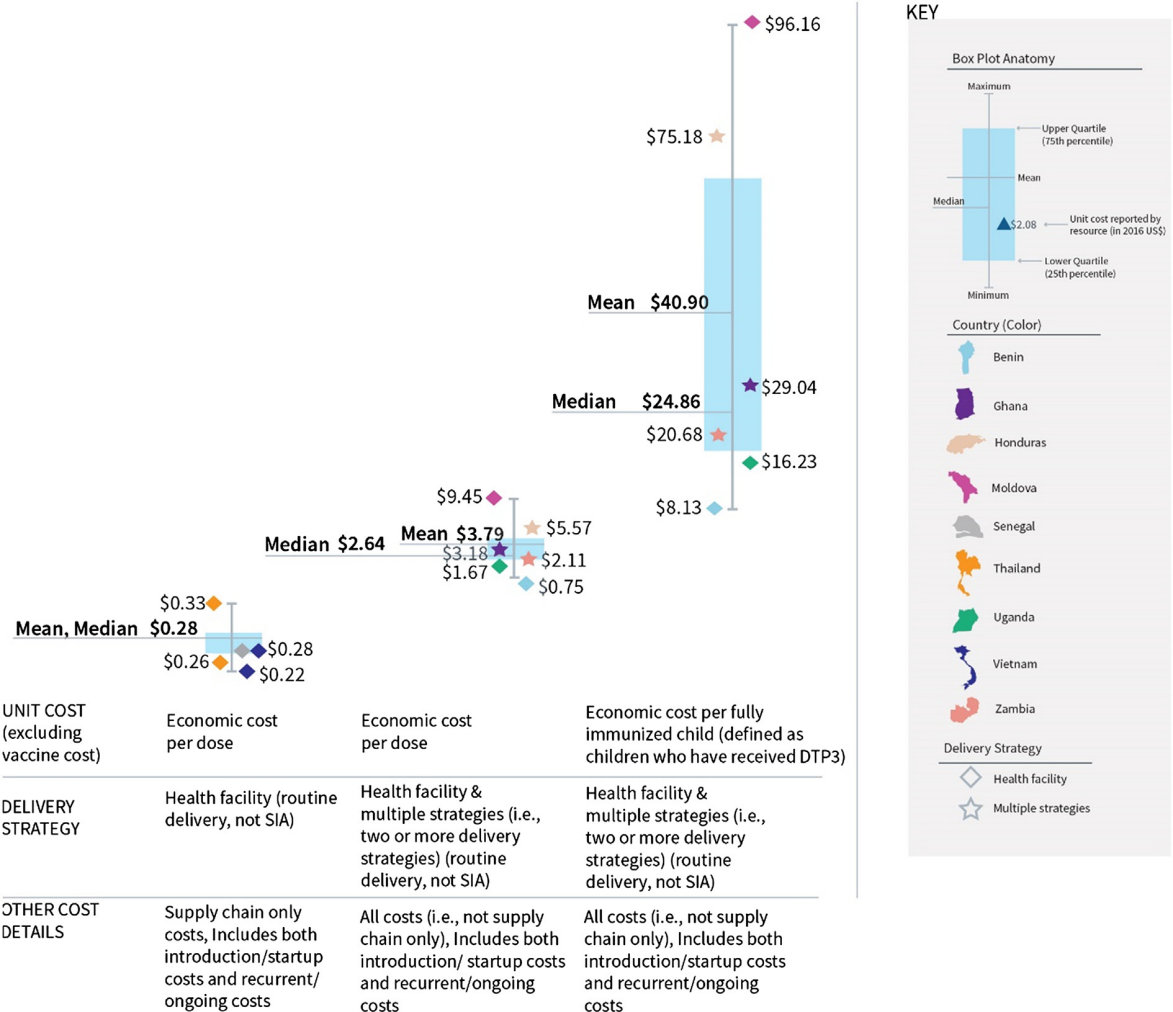
Full costs for delivering a schedule of vaccines, excluding vaccine costs (2016 US$).

Looking only at supply chain-related costs, the full, economic cost per dose of delivering vaccination schedules containing 6–7 antigens (excluding vaccine cost) ranged from $0.22 to $0.33 (Fig. 7, left boxplot; mean and median $0.28). Looking at all costs (i.e., not supply chain only), the full, economic cost per dose of delivering schedules of 4–8 vaccines to under one-year-olds ranged from $0.75 to $9.45, with a mean of $3.79 and median of $2.64 (Fig. 7, middle boxplot). This equates to a cost per fully immunized child (defined by the study authors as children who have received DTP3) ranging from $8.13 to $96.16, with a mean of $40.90 and median of $24.86 (Fig. 7, right boxplot). (See also Supplementary Appendix 7.)

## 5 Discussion

This systematic review responded to the need for comprehensive, easily accessible, and user-friendly evidence on immunization delivery unit costs in LMICs. It goes beyond past attempts that looked only at a subset of vaccines, a subset of economic evaluations, or new vaccine introduction only. It considered over 15,000 resources, ultimately drawing from 61 resources without focusing solely on a particular vaccine, delivery strategy, type of cost analysis, or setting. The resulting dataset includes 410 immunization delivery unit costs in 2016 US$.

Immunization programs have made great strides in LMICs, with increased uptake of new and underused vaccines and global vaccination coverage reported to be 86% [20]. Our review has shown that quality evidence on the cost of delivering vaccines is increasing though is still nascent. Unit costs are available from only 33 countries, primarily low income, many of which are Gavi-eligible or countries in transition from Gavi support. Funding support from donors and development partners for costing studies likely explains the larger body of cost evidence in Gavi-eligible or transitioning countries. It was outside the scope of our review to investigate how Gavi transition may affect the cost of delivering services, but this could be an interesting topic for further research.

The overwhelming majority of unit costs are for health facility-based delivery, despite the widespread use of outreach/mobile strategies and vaccination campaigns. Our review has also shown that we know relatively little about the costs of delivering single vaccines other than PCV and HPV, or the cost of delivering schedules of vaccines due to their diversity in composition and their number of antigens.

There is limited costing data from regions other than Sub-Saharan Africa, from upper-middle income countries and on the cost of delivering vaccines through SIAs and using non health facility-based strategies, such as outreach/mobile and schools [21]. Gaps in evidence also exist on the financial and fiscal cost of delivering vaccines. Addressing these gaps may be particularly helpful for country-level planners and policymakers who are likely to use these types of cost for planning and budgeting.

Some readers may find the cost ranges to be higher than current cost per dose estimates being used in many LMICs for planning or budgeting purposes [22]. The eight cost ranges we generated from comparable unit costs have produced useful, albeit wide, cost ranges for: (1) the delivery of single, newly introduced vaccines; (2) the introduction of HPV vaccine; and (3) the delivery of schedules of vaccines. However, limited comparable unit costs prevented us from generating cost ranges that would permit conclusions regarding the relative cost of delivery strategies, or the relative cost of vaccine delivery in countries at different income levels.

At times the findings are contradictory. Considering incremental costs, the economic cost per dose for delivery of single, newly introduced vaccines (i.e., PCV and Rotavirus) at health facilities ranged from $0.48 to 1.38. The economic cost per dose for delivery of HPV in health facilities and schools ranged from $1.95 to 2.24; the higher delivery cost may be related to pilot/demonstration and school-based delivery. When considering full costs, the economic cost per dose for delivery of a schedule of vaccines to children under one at health facilities and through multiple strategies ranged from $0.75 to $9.45. Country income level may explain some of the difference: the first range represents costs from low-income countries, the second and third ranges from low- and middle-income countries. In addition to country income level, the small number of unit costs that make up each range, comprised of different cost categories, coming from different settings (e.g., geography, pilot/demonstration vs. at-scale), and calculated using different methodologies may help explain these sometimes confusing findings.

The cost ranges for HPV vaccine (financial cost per dose from $1.95 to $2.24 for school-based delivery, and economic cost per dose from $2.42 to $4.29 for health facility and school-based delivery) used data from pilot/demonstration introduction of HPV. Cost data from pilot/demonstration introductions can inform countries that are considering new vaccine introductions, as new vaccines are often rolled out in phases and it is important to consider that smaller delivery volumes in these early roll-out periods often prevent economies of scale from being reached, resulting in higher unit costs. However, as many countries have now expanded the pilot/demonstration implementation of HPV, more research is needed to know whether these cost ranges remain valid for at-scale implementation. Interestingly, the mean and median economic cost for HPV vaccine delivery ($2.06 and $2.03) is two-thirds the financial cost of delivery ($3.30 and $3.02), although the former only considers school delivery and the latter both health facility and school delivery.

We chose to present cost ranges as opposed to cost benchmarks with single point estimates for two main reasons. First, given the limited number of comparable unit costs that comprise the ranges, we could not objectively say that a single point estimate should be used for comparison or evaluation of country performance (e.g., to understand if country costs are below, on target with, or above the mean or median), or used as a cost norm to represent “average” performance.

Second, presenting cost ranges and noting the individual unit costs that make up the range reflects our desire to highlight the cost variation across different contexts. The 410 unit costs are unique in their makeup, representing different types of costs, delivery strategies, vaccines, country contexts, and study methodologies. Our analysis did not attempt to explain this variation; exploring the data further may lend insight into the existing variation.

Notably, differences in the cost categories (e.g., human resources, transport, training, supplies, etc.) included in the unit costs also limited comparability. Alignment on cost category definitions and inclusion of all relevant cost categories (e.g., per diem and travel allowances, program management, and waste management) in unit cost calculations should be a priority to improve comparability across settings and account for the full range of costs.

Additionally, better guidance on methods for immunization costing and reporting of results is warranted; publications on both topics are anticipated in 2020.

The current findings are useful for those interested in using evidence on the cost of delivering immunization services to inform planning, budgeting, advocacy or research. This may include national and sub-national planners and policymakers, researchers, and international partners supporting country immunization and health system policy, planning, and financing. The IDC data can be used for costing studies, economic evaluations such as cost-effectiveness studies, budget impact analyses and other efforts. Some users may find it informative to consider the delivery costs together with the relevant vaccine price (Gavi subsidized or non-subsidized) in a particular setting; such calculations should take into account vaccine manufacturer, vial size and packaging, as well as freight charges, wastage rates and coverage assumptions.

## 6 Limitations

The major limitation of this work is that the dataset and analysis rely on the strength of each study’s methods and reporting as well as the quality and reliability of the underlying data. Limitations in reporting, such as lack of specificity about the costing methodology employed or study setting, may not reflect the quality of the actual costing study conducted. Known immunization data quality challenges undoubtedly influence the reported unit costs [23]. Specific to delivery strategies, it is likely that some costs reported by resources as being for “health facility” delivery also include delivery via outreach/mobile or other strategies.

Overall, we have taken a conservative approach to data extraction and interpretation, reporting only the language used by the author and preferring to report certain characteristics of the data as “not reported” or “unclear” rather than making inferences. We recognize that some misinterpretations of the reported data may have inadvertently occurred due to both limitations in reporting and human error of the research team. Further, by using the language as reported by authors even if likely erroneous (e.g., incorrect classification of economic costs, societal perspective, etc.), and not recoding information using standardized definitions, we may have slightly reduced the number of comparable unit costs for cost ranges.

The cost ranges are limited by the heterogeneity in the dataset, reflected in the small number of comparable unit costs that could be used to develop them. Additionally, wide ranges point to the large variability in the data. Modelling work to expand the IDC database is expected to be released in 2019, which may fill in data gaps, increase the number of comparable unit costs, allow development of additional cost ranges, and facilitate other comparisons and benchmarking.

Finally, we acknowledge that there are additional articles on the costs of immunization delivery that are not captured in our review due to our specific inclusion and exclusion criteria, and likely more grey literature from the reviewed time period that is not included. This approach also excluded some seminal resources on immunization costs published prior to 2005.

## 7 Conclusions

This review highlighted several gaps in global knowledge about the costs of delivering vaccines in low- and middle-income countries. With only six countries contributing almost two-thirds of the unit costs in the IDCC, additional research is warranted for a larger number of countries generally, and with emphasis on countries with middle income status and on geographies other than Sub-Saharan Africa. Future research that prioritizes costing a variety of delivery strategies and not solely health facility delivery would also contribute much to our understanding of delivery costs, particularly as more and more vaccines target older children and adults. Additionally, as campaigns are increasingly used to improve coverage and decrease morbidity and mortality from vaccine-preventable diseases, a better understanding of their costs, and the cost implications of integrating other services such as vitamin A supplementation or deworming tablets, could be helpful for planning and budgeting purposes.

Upcoming shifts in the global immunization agenda, such as a heightened focus on equity, integration of immunization with other primary healthcare services, and increased domestic funding for immunization as countries transition away from external support, may create the need for additional costing research. All future research should be much more explicit in reporting on methods, and following well-established economic principles and immunization costing guidelines, with an aim towards greater standardization to facilitate cost comparisons.
