# Supplementary material for: The costs of delivering vaccines in low- and middle-income countries: Findings from a systematic review
Source: Vaccine X. 2019 Jul 15;2:100034. doi: 10.1016/j.jvacx.2019.100034 (PMC6697256; doi:10.1016/j.jvacx.2019.100034)

## SupplementAry Appendix 1. search strategies and Yield

| **Search** | | **Query** | | **Items found** |
| --- | --- | --- | --- | --- |
| **PubMed** | | | | |
| #4 | #1 AND #2 AND #3 | | | **5,288** |
| #3 | delivery[tiab] OR campaign*[tiab] OR incremental[tiab] OR strategy[tiab] OR strategies[tiab] OR “cold chain”[tiab] OR logistic*[tiab] OR equipment[tiab] OR personnel[tiab] OR overhead[tiab] OR operational[tiab] OR transport*[tiab] OR distribut*[tiab] OR “non-vaccine”[tiab] OR “per dose”[tiab] OR “per capita”[tiab] OR “per child”[tiab] OR “per person”[tiab] OR “per fully immunized child”[tiab] OR “per fully immunised child”[tiab] OR “per FIC”[tiab] OR universal[tiab] OR “disability-adjusted”[tiab] OR DALY[tiab] OR extrapolat*[tiab] | | | 3,043,193 |
| #2 | immuniz*[tiab] OR immunis*[tiab] OR vaccin*[tiab] | | | 355,109 |
| #1 | "Costs and Cost Analysis"[mh] OR costs[tiab] OR cost effective*[tiab] OR costing[tiab] OR cost-benefit[tiab] OR cost-utility[tiab] OR "Immunization Programs/economics"[mh] OR "Vaccination/economics"[mh] OR "Mass Vaccination/economics"[mh] | | | 396,536 |
| **Embase** | | | **Items found: 7,912** | |
| #6 | #3 AND #4 AND #5 | | | |
| #5 | delivery:ti OR campaign*:ti OR incremental:ti OR strategy:ti OR strategies:ti OR 'cold chain':ti OR logistic*:ti OR equipment:ti OR personnel:ti OR overhead:ti OR operational:ti OR transport*:ti OR distribut*:ti OR 'non-vaccine':ti OR 'per dose':ti OR 'per capita':ti OR 'per child':ti OR 'per person':ti OR 'per fully immunized child':ti OR 'per fully immunised child':ti OR 'per fic':ti OR universal:ti OR 'disability-adjusted':ti OR daly:ti OR extrapolat*:ti OR delivery:ab OR campaign*:ab OR incremental:ab OR strategy:ab OR strategies:ab OR 'cold chain':ab OR logistic*:ab OR equipment:ab OR personnel:ab OR overhead:ab OR operational:ab OR transport*:ab OR distribut*:ab OR 'non-vaccine':ab OR 'per dose':ab OR 'per capita':ab OR 'per child':ab OR 'per person':ab OR 'per fully immunized child':ab OR 'per fully immunised child':ab OR 'per fic':ab OR universal:ab OR 'disability-adjusted':ab OR daly:ab OR extrapolat*:ab | | | |
| #4 | immunis*:ti OR immuniz*:ti OR vaccin*:ti OR immunis*:ab OR immuniz*:ab OR vaccin*:ab | | | |
| #3 | #1 OR #2 | | | |
| #2 | cost:ti OR costs:ti OR costing:ti OR cost:ab OR costs:ab OR costing:ab | | | |
| #1 | 'cost benefit analysis'/exp OR 'cost utility analysis'/exp OR 'cost effectiveness analysis'/exp | | | |

| **Search** | **Query** | | | **Items found** |
| --- | --- | --- | --- | --- |
| **Web of Science** | |  | | |
| #4 | #1 AND #2 AND #3 | | **6,194** | |
| #3 | TOPIC: (delivery OR campaign* OR incremental OR strategy OR strategies OR “cold chain” OR logistic* OR equipment OR personnel OR overhead OR operational OR transport* OR distribut* OR “non-vaccine” OR “per dose” OR “per capita” OR “per child” OR “per person” OR “per fully immunized child” OR “per fully immunised child” OR “per FIC” OR universal OR “disability-adjusted” OR DALY OR extrapolat*) | | | |
| #2 | TOPIC: (immuniz* OR immunis* OR vaccin*) | | | |
| #1 | TOPIC: (costs OR cost-effective* OR costing OR cost-benefit OR cost-utility) | | | |
| **EconLit** | | **151** | | |
| #4 | #1 AND #2 AND #3 | |  | |
| #3 | costs OR cost-effective* OR costing OR cost-benefit OR cost-utility | | | |
| #2 | delivery OR campaign* OR incremental OR strategy OR strategies OR “cold chain” OR logistic* OR equipment OR personnel OR overhead OR operational OR transport* OR distribut* OR “non-vaccine” OR “per dose” OR “per capita” OR “per child” OR “per person” OR “per fully immunized child” OR “per fully immunised child” OR “per FIC” OR universal OR “disability-adjusted” OR DALY OR extrapolat* | | | |
| #1 | immuniz* OR immunis* OR vaccin* | | | |
| **NHS EED** | | **169** | | |
| #4 | #1 AND #2 AND #3 | |  | |
| #3 | costs OR cost-effective* OR costing OR cost-benefit OR cost-utility | | | |
| #2 | delivery OR campaign* OR incremental OR strategy OR strategies OR “cold chain” OR logistic* OR equipment OR personnel OR overhead OR operational OR transport* OR distribut* OR “non-vaccine” OR “per dose” OR “per capita” OR “per child” OR “per person” OR “per fully immunized child” OR “per fully immunised child” OR “per FIC” OR universal OR “disability-adjusted” OR DALY OR extrapolat* | | | |
| #1 | immuniz* OR immunis* OR vaccin* | | | |
| **WHO Global Index Medicus** | | **17** | | |
| #4 | #1 AND #2 AND #3 | |  | |
| #3 | Filter: Health economic evaluations | | | |
| #2 | (tw:(immuniz* OR immunis* OR vaccin*)) | | | |
| #1 | (tw:(costs OR cost-effective* OR costing OR cost-benefit OR cost-utility)) | | | |

*Note:* WHO’s Global Index Medicus contains records from WHO regional indices for Africa, Latin America and the Caribbean, Middle East, Southeast Asia and Western Pacific, as well as WHO documents. Optional search of MEDLINE was not completed. Search string includes only two components due to database limitations. See http://www.globalhealthlibrary.net.

## Supplementary Appendix 2. Quality assessment

Below we describe the guiding principles, procedure and scoring for the quality assessment of each published article and grey literature resource.

### Principles

- We aimed for a parsimonious set of meaningful quality criteria, rather than a laundry list of items, to develop a rating for each resource. With the intention of striving for inclusion of costing resources that are most relevant, useful, and appropriate for country policymakers to use for planning and budgeting immunization programs, our criteria and scoring are somewhat forgiving. We aimed to identify “technically acceptable” resources for our target audience.
- Our set of quality criteria are based on a number of existing checklists: Global Health Cost Consortium 2017; Constenla et al. 2016; Avenir Health 2015; Husereau et al. 2013; Evers et al. 2005; Pegurri et al. 2005; Drummond et al. 1997.

### Procedure

- Questions are grouped in three areas: methodological rigor and reporting standards (8 items), uncertainty of results (3 items), and risk of bias and limitations (3 items) (Table 1). Extractors answered each of the questions about the resource at time of extraction based on the information filled out in the data extraction sheet.
- The quality scoring was quality reviewed as part of the data extraction tool, and proposed scoring changes were reviewed and agreed upon by the original extractor.
- One investigator reviewed all quality ratings against their respective data extraction and against that of the other reviewers, and adjusted scoring to ensure interrater reliability.

### Scoring

- Each item was given an individual score of 1 (lowest), 2, or 3 (highest); for some items there was also a “not applicable” option.
- Scores for all items were summed and averaged (with equal weighting of all categories and questions), excluding any “not applicable” answers, to produce a final score for each resource on the same 1 to 3 scale (Table 1).

### *Table 1. Quality assessment scoring*

| **Category** | **Area** | **Question** | **Scoring** |
| --- | --- | --- | --- |
| Methodology and reporting | Quality of input data/data source | Were primary data used for all cost data? (note: if primary data were not used at all the resource should be excluded) | 1 Partially  2 Mostly  3 Fully  n/a Source not reported (NR) |
|  | Sample strategy in relation to conclusions and generalizability | Were the conclusions and generalizability of findings appropriate given the sampling strategy? | 1 No  2 Mostly  3 Fully  n/a Conclusions not reported at all or for the costing portion of the study; sampling strategy not reported |
|  | Data analysis strategy | Were statistical tests used and confidence intervals (CIs) reported? | 1 No statistical test/CIs reported  2 Either statistical test or CI reported  3 Both reported |
|  | Allocation of shared costs | If shared costs were included, were methods for allocating them described? | 1 No/Shared costs excluded with no justification/shared costs not mentioned  2 Partly  3 Fully  n/a Shared costs excluded with justification |
|  | Annualization of capital costs including discount rate | Were capital items annualized using appropriate lifetimes and discount rates? | 1 No/Annualization of capital items excluded with no justification  2 Partly  3 Fully  n/a Annualization of capital items excluded with justification |
|  | Replicability | Were methods described well enough that the study could be replicated with the exact same results? | 1 No/methodology not reported  2 Partly  3 Fully |
|  |  | Was the purpose of the study clearly defined? | 1 No/purpose not reported  2 Partly  3 Fully |
|  | Reporting of results | Is the type of cost reported clear (economic, financial, fiscal; incremental, full)? | 1 No  2 Partly  3 Fully |
|  | Accuracy of reported findings | Does the sum of capital costs + recurrent costs equal reported total costs? | 1 No  2 Yes, for some of the findings reported  3 Yes, for all findings reported  n/a Capital or recurrent or total costs are NR |
|  | Accuracy of reported findings | Does the sum of all cost categories equal reported total costs? | 1 No, for all or most of the findings reported  2 Yes, for most of the findings reported  3 Yes, for all findings reported  n/a Cost categories and/or totals are NR |
| Uncertainty of results | Sensitivity analysis | If done, did the sensitivity analysis (SA) include all reasonable scenarios affecting costing results? | 1 No sensitivity analysis done  2 Partly  3 Fully  n/a SA was done, but is not applicable to delivery costs |
|  | Missing cost categories | Were all the important and relevant inputs identified and valued given the stated perspective? Are any relevant cost categories (line items or activities) missing that are not noted and justified as excluded? Is it clear what items are included in the unit costs? | 1 Yes, more than half are excluded/unclear  2 Yes, less than half are excluded/not completely clear  3 No, all/most of the relevant cost categories included and clear |
|  | Contextual factors | Are there any contextual factors related to the study setting that have not been accounted in the methods and/or results? | 1 Yes, some  3 No  n/a No contextual factors were reported |
| Risk of bias/ limitations | Author-stated limitations and/or possible areas of bias | Are limitations and potential sources of bias presented? If yes, do they make you question the findings? | 1 Presented and I fully question the findings / no limitations presented  2 Partially question the findings  3 Yes, but they don’t make me question the findings |
|  | Extractor-perceived limitations | Are there any extractor-perceived limitations that make you question the findings? | 1 Yes, and I fully question the findings  2 Some that make me partially question the findings  3 None, or yes but they don’t make me question the findings |

## SUPPLEMENTARY Appendix 3. Reference List

| **Reference** | **Unit Costs** | **Countries** | **Vaccines Costed*** |
| --- | --- | --- | --- |
| [Al-lela, O. Q. B., Bahari, M. B., Al-abbassi, M. G., Salih, M. R. M., & Basher, A. Y. (2012). Estimation of immunization providers’ activities cost, medication cost, and immunization dose errors cost in Iraq. *Vaccine*, 30(26), 3862–3866. https://doi.org/10.1016/j.vaccine.2012.04.014](https://doi.org/10.1016/j.vaccine.2012.04.014) | 2 | Iraq | BCG, Measles, MMR, HepB, DTP, OPV |
| [AMP. (2014). Costing and financing analyses of routine immunization and new vaccine introduction in Benin Final Report.**](https://static1.squarespace.com/static/556deb8ee4b08a534b8360e7/t/5596f9b2e4b00f5f813c3de5/1435957682517/FINALREPORT_BENIN.pdf) | 28 | Benin | BCG, Measles, DTP-HepB-Hib, OPV, PCV13, YF |
| [Ayieko, P., Griffiths, U. K., Ndiritu, M., Moisi, J., Mugoya, I. K., Kamau, T., … Scott, J. A. G. (2013). Assessment of Health Benefits and Cost-Effectiveness of 10-Valent and 13-Valent Pneumococcal Conjugate Vaccination in Kenyan Children. *PLoS ON*E, 8(6), 1–10. https://doi.org/10.1371/journal.pone.0067324](https://doi.org/10.1371/journal.pone.0067324) | 4 | Kenya | PCV10, PCV13 |
| Bem, J and Stewart, E. "Vaccine Costing Analysis Preliminary Results." Presentation at the Pharmaceutical Fund and Supply Agency, Addis Ababa, Ethiopia, September 2015. | 7 | Ethiopia | OPV, Rotavirus, (2 doses), TT, PCV10, BCG, Measles, DTP |
| [Bishai, D., Johns, B., Lefevre, A., & Nair, D. (2010). Cost effectiveness of measles eradication Final Report. Retrieved from http://www.who.int/immunization/sage/1_Bishai_Economic_analysis.pdf](http://www.who.int/immunization/sage/1_Bishai_Economic_analysis.pdf) | 2 | Uganda | Measles |
| [Brown, S. T., Schreiber, B., Cakouros, B. E., Wateska, A. R., Dicko, H. M., Connor, D. L., … Lee, B. Y. (2014). The benefits of redesigning Benin’s vaccine supply chain. *Vaccine*, 32(32), 4097–4103. https://doi.org/10.1016/j.vaccine.2014.04.090 ***](https://doi.org/10.1016/j.vaccine.2014.04.090) | 4 | Benin | BCG, Measles, TT, DTP-HepB-Hib, OPV, Rotavirus (2 doses), PCV13, YF |
| [Castañeda-Orjuela, C., Romero, M., Arce, P., Resch, S., Janusz, C. B., Toscano, C. M., & De la Hoz-Restrepo, F. (2013). Using standardized tools to improve immunization costing data for program planning: The cost of the Colombian Expanded Program on Immunization. *Vaccine*, 31(SUPPL.3), 72–79. https://doi.org/10.1016/j.vaccine.2013.05.038 †](https://doi.org/10.1016/j.vaccine.2013.05.038) | 4 | Colombia | BCG, MR, MMR, HepB, DTP, DT, Td, DTP-HepB-Hib, OPV, Rotavirus (2 doses), PCV7, YF, Influenza |
| [Cavailler, P., Lucas, M., Perroud, V., McChesney, M., Ampuero, S., Guérin, P. J., … Chaignat, C. L. (2006). Feasibility of a mass vaccination campaign using a two-dose oral cholera vaccine in an urban cholera-endemic setting in Mozambique. *Vaccine*, 24(22), 4890–4895. https://doi.org/10.1016/j.vaccine.2005.10.006](https://doi.org/10.1016/j.vaccine.2005.10.006) | 1 | Mozambique | OCV |
| [Chatterjee, S., Pant, M., Haldar, P., Aggarwal, M. K., & Laxminarayan, R. (2016). Current costs & projected financial needs of India’s universal immunization programme. *Indian Journal of Medical Research*, 143(JUNE), 801–808. https://doi.org/10.4103/0971-5916.192073](https://doi.org/10.4103/0971-5916.192073) | 2 | India | BCG, Measles, HepB, DTP, TT, OPV, JE |
| [Colombini, A., Badolo, O., Gessner, B. D., Jaillard, P., Seini, E., & Da Silva, A. (2011). Costs and impact of meningitis epidemics for the public health system in Burkina Faso. *Vaccine*, 29(33), 5474–5480. https://doi.org/10.1016/j.vaccine.2011.05.058](https://doi.org/10.1016/j.vaccine.2011.05.058) | 2 | Burkina Faso | Meningococcal |
| Dorji, K., Phuntsho, S., Pempa, Kumluang, S., Khuntha, S., Kulpeng, W., … Teerawattananon, Y. (2018). Towards the introduction of pneumococcal conjugate vaccines in Bhutan: A cost-utility analysis to determine the optimal policy option. Vaccine, 36(13), 1757-65. https://doi.org/10.1016/j.vaccine.2018.02.048 | 2 | Bhutan | PCV10, PCV13 |
| [Douba, A., Dagnan, S. N., Zengbe-Acray, P., Aka, J., & Lépri-Aka N. (2006). Perception du Programme élargi de vaccination (PEV) dans le district sanitaire de Bouna (Nord-Est de la Côte d ’ Ivoire). *Sante Publique*, 23(2), 113-121.](https://www.ncbi.nlm.nih.gov/pubmed/21896225) | 5 | Cote d'Ivoire | BCG, DTP-HepB, OPV |
| [Ebong, C. E., & Levy, P. (2011). Impact of the introduction of new vaccines and vaccine wastage rate on the cost-effectiveness of routine EPI: Lessons from a descriptive study in a Cameroonian health district. Co*st Effectiveness and Resource Allocation*, 9, 1–8. https://doi.org/10.1186/1478-7547-9-9](https://dx.doi.org/10.1186%2F1478-7547-9-9) | 3 | Cameroon | BCG, Measles, DTP-HepB-Hib, OPV, YF |
| [Goguadze, K., Chikovani, I., Gaberi, C., Maceira, D., Uchaneishvili, M., Chkhaidze, N., & Gotsadze, G. (2015). Costs of routine immunization services in Moldova: Findings of a facility-based costing study. *Vaccine*, 33(S1), A60–A65. https://doi.org/10.1016/j.vaccine.2014.12.034 **](https://doi.org/10.1016/j.vaccine.2014.12.034) | 4 | Moldova | BCG, MMR, HepB, DTP-HepB-Hib, OPV |
| [Gotsadze, G., Goguadze, K., Chikovani, I., & Maceira, D. (2014). Analyses of Costs and Financing of the Routine Immunization Program and New Vaccine Introduction in the Republic of Moldova Study Report. **](https://static1.squarespace.com/static/556deb8ee4b08a534b8360e7/t/5596fadee4b0402e04fe3d30/1435957982489/Moldova+EPIC+Final+Report+2014.pdf) | 34 | Moldova | BCG, MMR, HepB, DTP, DT, Td, DTwP-Hib, DTP-HepB-Hib, OPV, Rotavirus (2 doses) |
| [Griffiths, U. K., Hutton, G., & Das Dores Pascoal, E. (2005). The cost-effectiveness of introducing hepatitis B vaccine into infant immunization services in Mozambique. H*ealth Policy and Planning*, 20(1), 50–59. https://doi.org/10.1093/heapol/czi006](https://doi.org/10.1093/heapol/czi006) | 3 | Mozambique | BCG, Measles, TT, OPV, HepB, DTP-HepB |

| **Reference** | **Unit Costs** | **Countries** | **Vaccines Costed*** |
| --- | --- | --- | --- |
| [Griffiths, U. K., Korczak, V. S., Ayalew, D., & Yigzaw, A. (2009). Incremental system costs of introducing combined DTwP-hepatitis B-Hib vaccine into national immunization services in Ethiopia. *Vaccine*, 27(9), 1426–1432. https://doi.org/10.1016/j.vaccine.2008.12.037](https://doi.org/10.1016/j.vaccine.2008.12.037) | 2 | Ethiopia | DTwP-HepB-Hib |
| [Griffiths, U. K., Santos, A. C., Nundy, N., Jacoby, E., & Matthias, D. (2011). Incremental costs of introducing jet injection technology for delivery of routine childhood vaccinations: Comparative analysis from Brazil, India, and South Africa. *Vaccine*, 29(5), 969–975. https://doi.org/10.1016/j.vaccine.2010.11.038](https://doi.org/10.1016/j.vaccine.2010.11.038) | 15 | Brazil, India, South Africa | BCG, MMR, HepB, DTwP-Hib, YF, Measles, DT, TT, DTaP-Hib-IPV, PCV7, DTwP |
| [Griffiths, U. K., Bozzani, F. M., Chansa, C., Kinghorn, A., Kalesha-Masumbu, P., Rudd, C., … Schutte, C. (2016). Costs of introducing pneumococcal, rotavirus and a second dose of measles vaccine into the Zambian immunisation programme: Are expansions sustainable? *Vaccine*, 34(35), 4213–4220. https://doi.org/10.1016/j.vaccine.2016.06.050**](https://doi.org/10.1016/j.vaccine.2016.06.050) | 19 | Zambia | Measles, Rotavirus (2 doses), PCV10 |
| [Guthrie, T., Zikusooka, C., Kwesiga, B., Abewe, C., Lagony, S., Schutte, C., … Kinghorn, A. (2014). Costing and Financing Analyses of Routine Immunization in Uganda. Retrieved from https://static1.squarespace.com/static/556deb8ee4b08a534b8360e7/t/5596fa4ae4b07b7dda4dd04d/1435957834829/UGANDA+Immunization+Costing+Report+1+December+14+submitted+FINAL+update+15+12+14+errors.pdf**](https://static1.squarespace.com/static/556deb8ee4b08a534b8360e7/t/5596fa4ae4b07b7dda4dd04d/1435957834829/UGANDA+Immunization+Costing+Report+1+December+14+submitted+FINAL+update+15+12+14+errors.pdf) | 72 | Uganda | BCG, Measles, TT, DTP-HepB-Hib, OPV, PCV10 |
| [Haidari, L. A., Wahl, B., Brown, S. T., Privor-Dumm, L., Wallman-Stokes, C., Gorham, K., … Lee, B. Y. (2015). One size does not fit all: The impact of primary vaccine container size on vaccine distribution and delivery. *Vaccine*, 33(28), 3242–3247. https://doi.org/10.1016/j.vaccine.2015.04.018***](https://doi.org/10.1016/j.vaccine.2015.04.018) | 2 | Benin | BCG, Measles, TT, DTP-HepB-Hib, OPV, Rotavirus (2 doses), PCV13, YF |
| [Haidari, L. A., Brown, S. T., Ferguson, M., Bancroft, E., Spiker, M., Wilcox, A., … Lee, B. Y. (2016). The economic and operational value of using drones to transport vaccines. *Vaccine*, 34(34), 4062–4067. https://doi.org/10.1016/j.vaccine.2016.06.022***](https://doi.org/10.1016/j.vaccine.2016.06.022) | 1 | Mozambique | BCG, Measles, TT, DTP-HepB-Hib, OPV, PCV10 |
| [Haque, M., Waheed, M., & et al. (2016). The Pakistan Expanded Program on Immunization and the National Immunization Support Project. Retrieved from http://documents.worldbank.org/curated/en/264971484109785001/pdf/111815-WP-PAKImmunizationEA-PUBLIC.pdf](http://documents.worldbank.org/curated/en/264971484109785001/pdf/111815-WP-PAKImmunizationEA-PUBLIC.pdf) | 1 | Pakistan | BCG, Measles, DTP, OPV |
| Huang, X.X., Guillermet, E., Le Gargasson, J.B., Alfa, D.A., Gboja, R., Sossou, A.J., Jaillard, P. (2017). Costing analysis and anthropological assessment of the vaccine supply chain system redesign in the Comé District (Benin). Vaccine, 35(16), 2183-8. https://doi.org/10.1016/j.vaccine.2016.12.075 | 2 | Benin | BCG, Measles, TT, DTwP-HepB-Hib, PCV13, YF, OCV |
| [Hutubessy, R., Levin, A., Wang, S., Morgan, W., Ally, M., John, T., & Broutet, N. (2012). A case study using the United Republic of Tanzania: Costing nationwide HPV vaccine delivery using the WHO Cervical Cancer Prevention and Control Costing Tool. *BMC Medicine*, 10, 1–10. https://doi.org/10.1186/1741-7015-10-136 ††](https://doi.org/10.1186/1741-7015-10-136) | 14 | Tanzania | HPV |
| [Janusz, C. B., Castañeda-Orjuela, C., Molina Aguilera, I. B., Felix Garcia, A. G., Mendoza, L., Díaz, I. Y., & Resch, S. C. (2015). Examining the cost of delivering routine immunization in Honduras. *Vaccine*, 33(S1), A53–A59. https://doi.org/10.1016/j.vaccine.2015.01.016 **](https://doi.org/10.1016/j.vaccine.2015.01.016) | 2 | Honduras | BCG, MMR, HepB, Td, DTP-HepB-Hib, OPV, IPV, Rotavirus (2 doses), PCV13, YF, Influenza |
| [Kar, S. K., Sah, B., Patnaik, B., Kim, Y. H., Kerketta, A. S., Shin, S., … Wierzba, T. F. (2014). Mass Vaccination with a New, Less Expensive Oral Cholera Vaccine Using Public Health Infrastructure in India: The Odisha Model. *PLoS Neglected Tropical Diseases,* 8(2). https://doi.org/10.1371/journal.pntd.0002629](https://doi.org/10.1371/journal.pntd.0002629) | 3 | India | OCV |
| [Kaucley, L., & Levy, P. (2015). Cost-effectiveness analysis of routine immunization and supplementary immunization activity for measles in a health district of Benin. *Cost Effectiveness and Resource Allocation*, 13(1), 14. https://doi.org/10.1186/s12962-015-0039-7](https://doi.org/10.1186/s12962-015-0039-7) | 6 | Benin | Measles |
| [Khan, I. A., Saha, A., Chowdhury, F., Khan, A. I., Uddin, M. J., Begum, Y. A., … Qadri, F. (2013). Coverage and cost of a large oral cholera vaccination program in a high-risk cholera endemic urban population in Dhaka, Bangladesh. *Vaccine*, 31(51), 6058–6064. https://doi.org/10.1016/j.vaccine.2013.10.021 †††](https://doi.org/10.1016/j.vaccine.2013.10.021) | 3 | Bangladesh | OCV |
| [Le Gargasson, J. B., Nyonator, F. K., Adibo, M., Gessner, B. D., & Colombini, A. (2015). Costs of routine immunization and the introduction of new and underutilized vaccines in Ghana. *Vaccine*, 33(S1), A40–A46. https://doi.org/10.1016/j.vaccine.2014.12.081**](https://doi.org/10.1016/j.vaccine.2014.12.081) | 4 | Ghana | BCG, Measles, TT, DTP-HepB-Hib, OPV, YF, Rotavirus (2 doses), PCV13 |
| [Levin, C. E., Nelson, C. M., Widjaya, A., Moniaga, V., & Anwar, C. (2005). Costs of hepatitis B vaccine in a prefilled syringe in Indonesia. *Bulletin of the World Health Organization*, 83(3), 456–461. https://doi.org//S0042-96862005000600014](https://doi.org/S0042-96862005000600014) | 2 | Indonesia | HepB |
| [Levin, C. E., Van Minh, H., Odaga, J., Rout, S. S., Ngoc, D. N. T., Menezes, L., … LaMontagne, D. S. (2013). Delivery cost of human papillomavirus vaccination of young adolescent girls in Peru, Uganda and Viet Nam. *Bulletin of the World Health Organization*, 91(8), 585–592. https://doi.org/10.2471/BLT.12.113837](https://doi.org/10.2471/BLT.12.113837) | 10 | Peru, Uganda, Vietnam | HPV |

| **Reference** | **Unit Costs** | **Countries** | **Vaccines Costed*** |
| --- | --- | --- | --- |
| [Levin, A., Wang, S. A., Levin, C., Tsu, V., & Hutubessy, R. (2014). Costs of introducing and delivering HPV vaccines in low and lower middle income countries: Inputs for GAVI policy on introduction grant support to countries. *PLoS ONE*, 9(6). https://doi.org/10.1371/journal.pone.0101114](https://doi.org/10.1371/journal.pone.0101114) | 30 | Bhutan, India, Peru, Tanzania, Uganda, Vietnam | HPV |
| Lydon, P., Zipursky, S., Tevi-Benissan, C., Djingarey, M.H., Gbedonou, P., Youssouf, B.O., Zaffran, M. (2014). Economic benefits of keeping vaccines at ambient temperature during mass vaccination: the case of meningitis A vaccine in Chad. Bulletin of the World Health Organization, 92, 86-92. http://dx.doi.org/10.2471/BLT.13.123471 | 2 | Chad | Meningococcal |
| [Mascareñas, A., Salinas, J., Tasset-Tisseau, A., Mascareñas, C., & Khan, M. M. (2005). Polio immunization policy in Mexico: Economic assessment of current practice and future alternatives. *Public Health*, 119(6), 542–549. https://doi.org/10.1016/j.puhe.2004.08.020](https://doi.org/10.1016/j.puhe.2004.08.020) | 2 | Mexico | OPV |
| Minh, H.V., My, N.T.T., & Jit, M. (2017). Cervical cancer treatment costs and cost-effectiveness analysis of human papillomavirus vaccination in Vietnam: a PRIME modeling study. BMC Health Services Research, 17, 353. https://doi.org/10.1186/s12913-017-2297-x | 1 | Vietnam | HPV |
| [Minh, V. H., Nguyen, T. B. Y., Bao, G. K., Lan, H. D., Thuy, H. N., & Wright, P. (2008). Cost of providing the expanded programme on immunization: Findings from a facility-based study in Viet Nam, 2005. B*ulletin of the World Health Organization*, 86(6), 429–434. https://doi.org/10.2471/BLT.07.045161](https://doi.org/10.2471/BLT.07.045161) | 6 | Vietnam | BCG, Measles, HepB, DTP, TT, OPV, JE |
| [Moodley, I., Tathiah, N., & Sartorius, B. (2016). The costs of delivering human papillomavirus vaccination to Grade 4 learners in KwaZulu-Natal, South Africa. *South African Medical Journal* = Suid-Afrikaanse Tydskrif Vir Geneeskunde, 106(5), 60. https://doi.org/https://dx.doi.org/10.7196/SAMJ.2016.v106i5.9988](http://dx.doi.org/10.7196/samj.2016.v106i5.9988) | 1 | South Africa | HPV |
| [Mvundura, M., Kien, V. D., Nga, N. T., Robertson, J., Van Cuong, N., Tung, H. T., … Levin, C. (2014). How much does it cost to get a dose of vaccine to the service delivery locationα Empirical evidence from Vietnam’s Expanded Program on Immunization. *Vaccine*, 32(7), 834–838. https://doi.org/10.1016/j.vaccine.2013.12.029 ‡](https://doi.org/10.1016/j.vaccine.2013.12.029) | 4 | Vietnam | BCG, Measles, HepB, DTwP, TT, OPV, DTP-HepB-Hib |
| [Mvundura, M., Lorenson, K., Chweya, A., Kigadye, R., Bartholomew, K., Makame, M., … Kristensen, D. (2015). Estimating the costs of the vaccine supply chain and service delivery for selected districts in Kenya and Tanzania. *Vaccine*, 33(23), 2697–2703. https://doi.org/10.1016/j.vaccine.2015.03.084](https://doi.org/10.1016/j.vaccine.2015.03.084) | 2 | Kenya, Tanzania | BCG, Measles, Td, DTP-HepB-Hib, OPV, PCV10, YF |

| **Reference** | **Unit Costs** | **Countries** | **Vaccines Costed*** |
| --- | --- | --- | --- |
| Mvundura, M., Lydon, P., Gueye, A., Diaw, I.K., Landoh, D.E., Toi, B., … Kristensen, D. (2017). An economic evaluation of the controlled temperature chain approach for vaccine logistics: evidence from a study conducted during a meningitis A vaccine campaign in Togo. The Pan African Medical Journal, 27(Supp 3), 27. doi:10.11604/pamj.supp.2017.27.3.12087 | 2 | Togo | Meningococcal |
| [Ngabo, F., Levin, A., Wang, S. A., Gatera, M., Rugambwa, C., Kayonga, C., … Hutubessy, R. (2015). A cost comparison of introducing and delivering pneumococcal, rotavirus and human papillomavirus vaccines in Rwanda. *Vaccine*, 33(51), 7357–7363. https://doi.org/10.1016/j.vaccine.2015.10.022](https://doi.org/10.1016/j.vaccine.2015.10.022) | 24 | Rwanda | PCV7, Rotavirus (3 doses), HPV |
| [Pan American Health Organization. (2014). Comprehensive costing and financial flows analysis of the national immunization program in Honduras, 2011. **](https://static1.squarespace.com/static/556deb8ee4b08a534b8360e7/t/5596fe06e4b00d130d557c2b/1435958790966/MERGED_HONDURAS.pdf) | 1 | Honduras | BCG, MMR, HepB, DTP, Td, DTP-HepB-Hib, OPV, IPV, Rotavirus (2 doses), PCV13, YF, Influenza |
| [PATH. (2013). Optimize: Senegal Report. ‡](http://www.path.org/publications/files/TS_opt_senegal_rpt.pdf) | 1 | Senegal | BCG, Measles, TT, DTwP-HepB-Hib, OPV, YF |
| [PATH, World Health Organisation, Health Systems Research Institute, & Mahidol University. (2011). An Assessment of Vaccine Supply Chain and Logistics Systems in Thailand. Path, (September), 1–58. ‡](https://www.path.org/publications/files/TS_opt_vac_sup_thai.pdf) | 2 | Thailand | BCG, Measles, MMR, HepB, DTP, OPV, JE, |
| [Quentin, W., Terris-Prestholt, F., Changalucha, J., Soteli, S., Edmunds, W. J., Hutubessy, R., … Watson-Jones, D. (2012). Costs of delivering human papillomavirus vaccination to schoolgirls in Mwanza Region, Tanzania. *BMC Medicine*, 10(November 2011). https://doi.org/10.1186/1741-7015-10-137](https://doi.org/10.1186/1741-7015-10-137) | 5 | Tanzania | HPV |
| Riewpaiboon, A., Sooksriwong, C., Chaiyakunapruk, N., Tharmaphornpilas, P., Techathawat, S., Rookkapan, K., … Suraratdecha, C. (2015). Optimizing national immunization program supply chain management in Thailand: an economic analysis. Public Health, 129(7), 899-906. 1016/j.puhe.2015.04.016 | 4 | Thailand | BCG, Measles, MMR, HepB, DTP, DT, DTP-Hib, OPV, JE |
| Routh, J.A., Sreenivasan, N., Adhikari, B.B., Andrecy, L.L., Bernateau, M., Abimbola, T., … Mintz, E.D. (2017). Cost evaluation of a government-conducted oral cholera vaccination campaign - Haiti, 2013. The American Society of Tropical Medicine and Hygiene, 97(4), 37-42. doi:10.4269/ajtmh.16-1023 | 6 | Haiti | OCV |
| [Ruhago, G. M., Ngalesoni, F. N., Robberstad, B., & Norheim, O. F. (2015). Cost-effectiveness of live oral attenuated human rotavirus vaccine in Tanzania. *Cost Effectiveness and Resource Allocation*, 13(1), 1–12. https://doi.org/10.1186/s12962-015-0033-0](https://doi.org/10.1186/s12962-015-0033-0) | 3 | Tanzania | Rotavirus (2 doses) |
| [Sarker, A. R., Islam, Z., Khan, I. A., Saha, A., Chowdhury, F., Khan, A. I., … Khan, J. A. M. (2015). Estimating the cost of cholera-vaccine delivery from the societal point of view: A case of introduction of cholera vaccine in Bangladesh. *Vaccine*, 33(38), 4916–4921. https://doi.org/10.1016/j.vaccine.2015.07.042 †††](https://doi.org/10.1016/j.vaccine.2015.07.042) | 3 | Bangladesh | OCV |
| [Schaetti, C., Weiss, M. G., Ali, S. M., Chaignat, C. L., Khatib, A. M., Reyburn, R., … Hutubessy, R. (2012). Costs of Illness Due to Cholera, Costs of Immunization and Cost-Effectiveness of an Oral Cholera Mass Vaccination Campaign in Zanzibar. *PLoS Neglected Tropical Diseases*, 6(10). https://doi.org/10.1371/journal.pntd.0001844](https://doi.org/10.1371/journal.pntd.0001844) | 2 | Tanzania | OCV |
| [Schütte, C., Chansa, C., Marinda, E., Guthrie, T. A., Banda, S., Nombewu, Z., … Kinghorn, A. (2015). Cost analysis of routine immunisation in Zambia. *Vaccine*, 33(S1), A47–A52. https://doi.org/10.1016/j.vaccine.2014.12.040**](https://doi.org/10.1016/j.vaccine.2014.12.040) | 4 | Zambia | BCG, Measles, DTP-HepB-Hib, OPV |
| Suharlim, C. and Menzies, N. (2018) Personal communication, based on [EPI Costing and Financing Study - Phase 2 (EPIC2) Dataverse](https://dataverse.harvard.edu/dataverse/EPIC2) (Harvard T.H. Chan School of Public Health). https://dataverse.harvard.edu/dataverse/EPIC2 | 16 | Benin, Ghana, Honduras, Moldova, Uganda, Zambia | BCG, Measles, MMR, HepB, DTP, Td, DTP-Hib, DTP-HepB-Hib, OPV, IPV, Rotavirus (2 doses), PCV13, YF |
| [Sume, G. E., Fouda, A. A. B., Kobela, M., Nguelé, S., Emah, I., & Atem, P. (2013). A locally initiated and executed measles outbreak response immunization campaign in the nylon health district, Douala Cameroon 2011. *BMC Research Notes*, 6(1). https://doi.org/10.1186/1756-0500-6-100](https://doi.org/10.1186/1756-0500-6-100) | 1 | Cameroon | Measles |
| Teshome, S., Desai, S., Kim, J.H., Belay, D., & Mogasale, V. (2018). Feasibility and costs of a targeted cholera vaccination campaign in Ethiopia. Human Vaccines & Immunotherapeutics. https://doi.org/10.1080/21645515.2018.1460295 | 5 | Ethiopia | OCV |
| [Usuf, E., Mackenzie, G., Lowe-Jallow, Y., Boye, B., Atherly, D., Suraratdecha, C., & Griffiths, U. K. (2014). Costs of vaccine delivery in the Gambia before and after, pentavalent and pneumococcal conjugate vaccine introductions. *Vaccine*, 32(17), 1975–1981. https://doi.org/10.1016/j.vaccine.2014.01.045](https://doi.org/10.1016/j.vaccine.2014.01.045) | 4 | The Gambia | DTP-HepB-Hib, HepB, TT, OPV, YF , PCV7 |
| [VillageReach. (2009). Comparison of Costs Incurred in Dedicated and Diffused Vaccine Logistics Systems, (October), 1–42. ‡‡‡](http://www.villagereach.org/wp-content/uploads/2010/10/091009-VillageReach-Cost-Study-Report.pdf) | 6 | Mozambique | BCG, Measles, TT, DTP-HepB, OPV |
| [Yin, Z., Beeler Asay, G. R., Zhang, L., Li, Y., Zuo, S., Hutin, Y. J., … Jiang, F. (2012). An economic evaluation of the use of Japanese encephalitis vaccine in the expanded program of immunization of Guizhou province, China. *Vaccine*, 30(37), 5569–5577. https://doi.org/10.1016/j.vaccine.2012.05.068](https://doi.org/10.1016/j.vaccine.2012.05.068) | 2 | China | JE |
| Yu, W., Lu, M., Wang, H., Rodewald, L., Ji, S., Ma, C., … Liu, Y. (2018). Routine immunization services costs and financing in China, 2015. Vaccine, 36(21), 3041-7. 10.1016/j.vaccine.2018.04.008 | 4 | China | BCG, MR, MMR, HepB, DTP, DT, OPV, IPV, JE, Meningococcal |
| [Zengbe-Acray, P., Douba, A., Traore, Y., Dagnan, S., Attoh-Toure, H., & Ekra, D. (2009). Coûts de la riposte vaccinale contre la fièvre jaune à abidjan, 2001. *Sante Publique*, 21(4), 383–391.](https://www.cairn.info/revue-sante-publique-2009-4-page-383.htm) | 2 | Cote d'Ivoire | YF |

*DT = Diphtheria and tetanus toxoids, pediatric formulation; DTaP = Diphtheria and tetanus toxoids and acellular pertussis vaccine, pediatric formulation; DTP = Diphtheria and tetanus toxoids and whole-cell pertussis vaccine, pediatric formulation; HepB = Hepatitis B Vaccine; Hib = Haemophilus influenzae type b; HPV = Human Papillomavirus; IPV = Inactivated Poliovirus Vaccine; JE = Japanese Encephalitis; MCV = Measles antigen-containing vaccines; MMR = Measles, Mumps & Rubella Vaccine; MR = Measles-rubella Vaccine; OPV = Oral Polio Vaccine; PCV = Pneumococcal Conjugate Vaccine; PCV7 = Pneumococcal Conjugate Vaccine (7-valent); PCV10 = Pneumococcal Conjugate Vaccine (10-valent); PCV13 = Pneumococcal Conjugate Vaccine (13-valent); Tetanus & diphtheria Vaccine, adult/adolescent formulation; TT = Tetanus Toxoid; YF = Yellow Fever

** EPI Costing and Financing Project (EPIC)

*** Highly Extensible Resource for Modeling Event-driven Supply chains (HERMES) Framework

† ProVac Initiative

†† Cervical Cancer Prevention and Control Costing (C4P) tool demonstration for planning and costing nationwide HPV vaccination programs

††† Introduction of Cholera Vaccine in Bangladesh (ICVB)

 ‡ Project Optimize

‡‡ HPV Vaccines: Evidence for Impact project

‡‡‡ Project to Support PAV (EPI) to strengthen the management, reliability and quality of the health system in Mozambique

## Supplementary Appendix 4. Cost Category Inclusion in Immunization Delivery Unit Costs

| Cost category | Economic unit costs (%) | Financial unit costs (%) | Fiscal unit costs (%) |
| --- | --- | --- | --- |
| Paid human resources | 90 | 54 | 100 |
| Volunteer human resources | 17 | 2 | 0 |
| Per diem & travel allowances | 59 | 59 | 48 |
| Cold chain equipment & overheads | 91 | 77 | 100 |
| Vehicles, transport & fuel | 98 | 98 | 100 |
| Program management | 40 | 55 | 22 |
| Training & capacity building | 71 | 89 | 87 |
| Social mobilization & advocacy | 69 | 91 | 87 |
| Adverse event monitoring | 23 | 28 | 0 |
| Buildings, utilities, other overheads & shared costs | 57 | 32 | 100 |
| Vaccines | 83 | 64 | 91 |
| Vaccine supplies | 75 | 85 | 91 |
| Waste management | 38 | 68 | 52 |
| Other supplies & recurrent costs | 51 | 44 | 100 |
| Other category (non-vaccine) costs | 59 | 30 | 39 |

## Supplementary Appendix 5. Incremental cost Range for single, newly introduced vaccines, Excluding Vaccine Cost (2016 US$)

The table below further details Figure 5. We estimate the incremental cost per dose to deliver a single, newly introduced vaccine such as PCV or Rotavirus (3-dose) at health facilities in low-income countries to be $0.48 to $1.38 (economic costs) and $0.16 to $2.54 (economic, financial, and fiscal costs). Full immunization (three doses) ranges from $1.45 to $4.20 (economic costs).


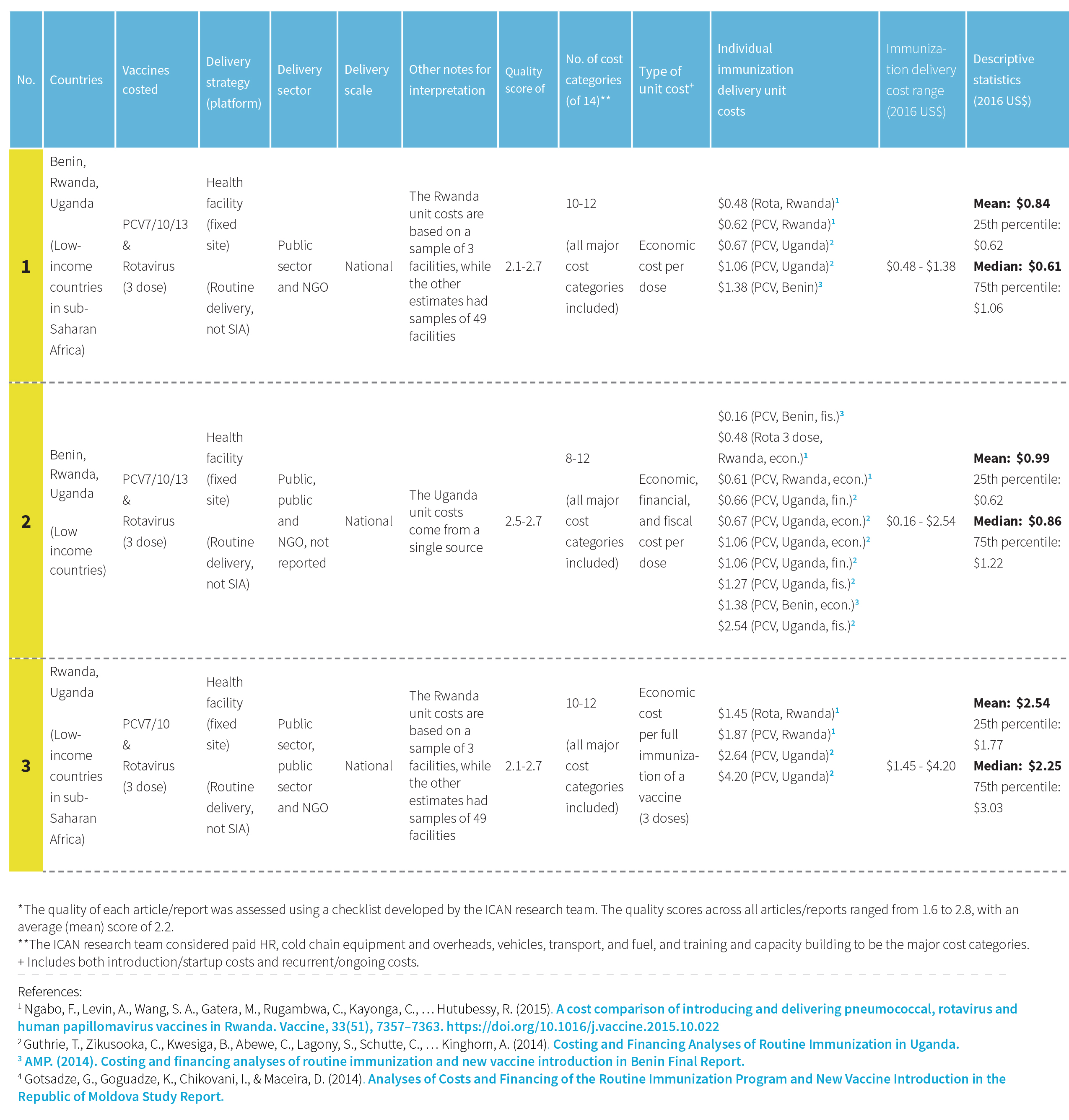


# Supplementary Appendix 6. Incremental Cost Range for Introducing HPV Vaccine to an Existing Schedule, Excluding Vaccine Cost (2016 US$)

The table below further details Figure 6 and depicts the cost of introducing HPV vaccine. Introducing HPV at schools on a pilot/project basis costs $1.95 to $4.29 (incremental costs). The lower end of the range represents financial costs, shown by cost range number 4, whereas the higher end of the range corresponds with economic costs, shown by cost range number 5.


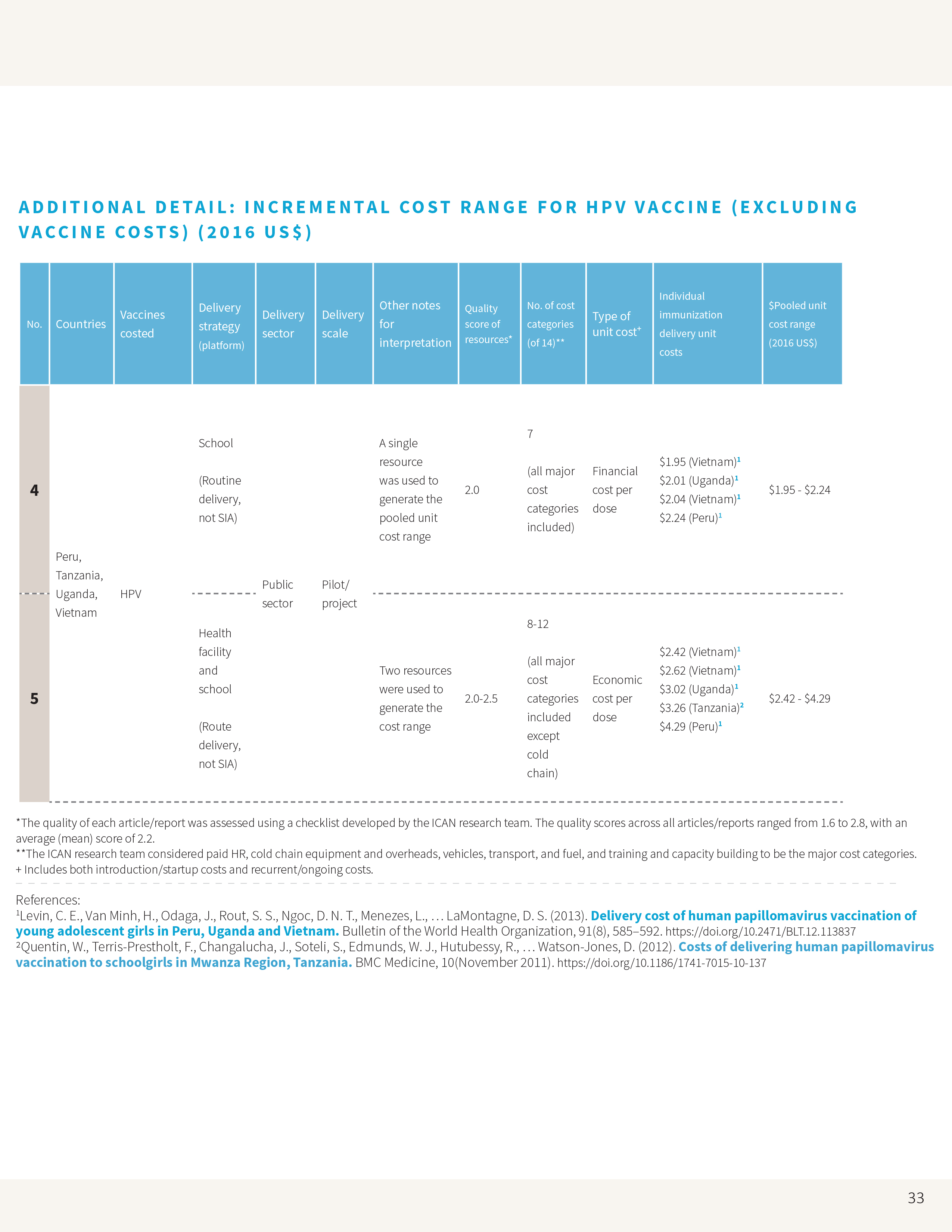


## Supplementary Appendix 7. Full costs for delivering a schedule of vaccines, Excluding Vaccine Cost (2016 US$)

The table below further details Figure 7 and depicts the full costs for delivering schedules of vaccines. Considering only supply chain-related costs, the full, economic cost per dose of delivering vaccination schedules containing 6 to 7 antigens range from $0.22 to $0.33. We estimate the costs (i.e., all costs, not supply chain only) of delivering a schedule of four to eight antigens for under one-year-olds to range from $0.75 to $9.45 (full, economic costs). This equates to a cost per fully immunized child (children who have received DTP3) of $8.13 to $96.16.


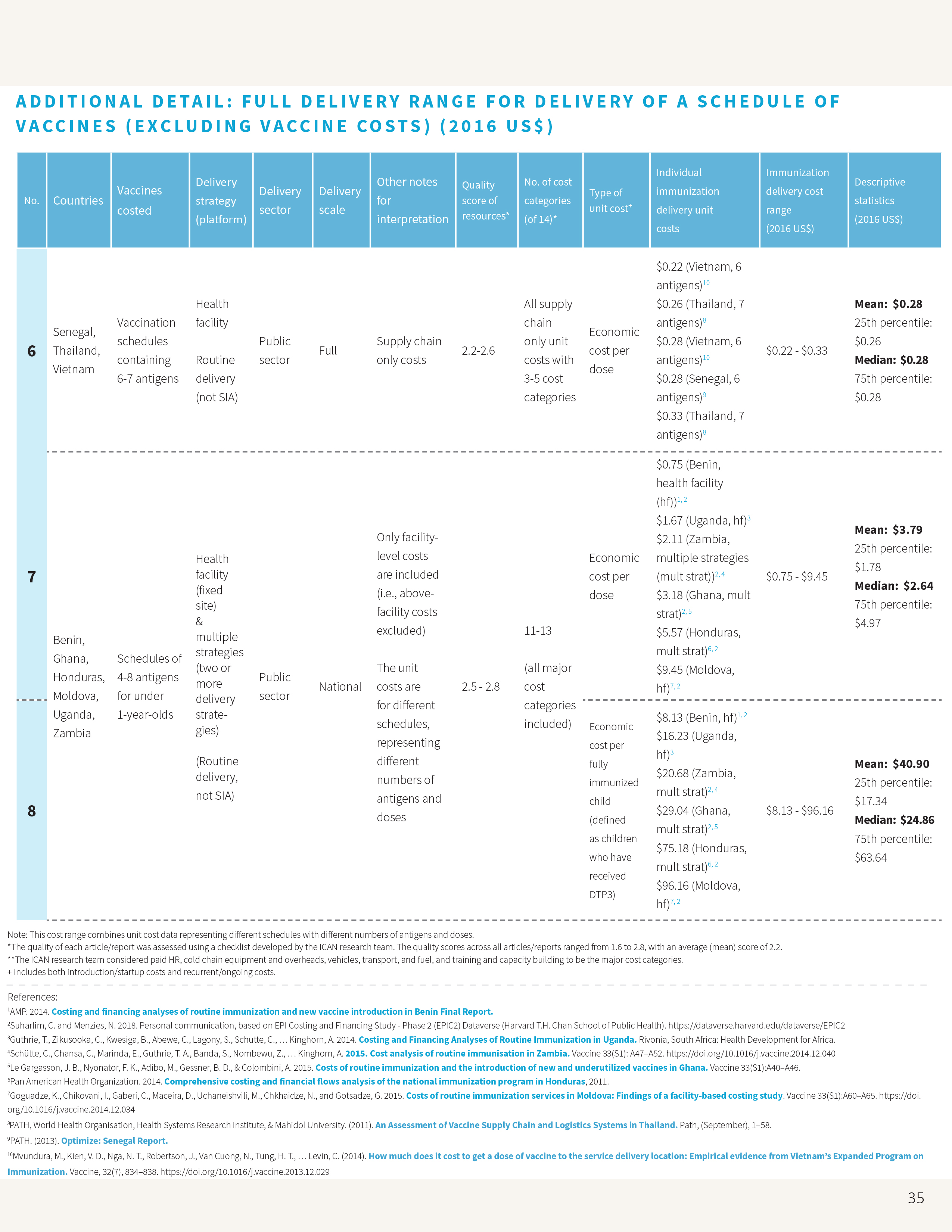

Supplement: Supplementary data 1 [file mmc1.docx]
